# Supplementing honey bee (Hymenoptera: Apidae) colonies with pollen increases their pollinating activity on nectariferous crops with anthers isolated from stigmas

**DOI:** 10.1093/jee/toad222

**Published:** 2023-12-13

**Authors:** Stan Chabert, Nicolas Morison, Marie-Josée Buffière, Laurent Guilbaud, Céline Pleindoux, Géraud de Premorel, Philippe Royer, Marie Harruis, Bernard E Vaissière

**Affiliations:** UR406 Abeilles et Environnement, Institut National de Recherche pour l’Agriculture, l’alimentation et l’Environnement (INRAE), Site Agroparc, Domaine Saint-Paul, CS 40509, 84914 Avignon Cedex 9, France; Department of Entomology and Nematology, University of Florida, 1881 Natural Area Drive, Gainesville, FL 32611, USA; UR406 Abeilles et Environnement, Institut National de Recherche pour l’Agriculture, l’alimentation et l’Environnement (INRAE), Site Agroparc, Domaine Saint-Paul, CS 40509, 84914 Avignon Cedex 9, France; UR406 Abeilles et Environnement, Institut National de Recherche pour l’Agriculture, l’alimentation et l’Environnement (INRAE), Site Agroparc, Domaine Saint-Paul, CS 40509, 84914 Avignon Cedex 9, France; UR406 Abeilles et Environnement, Institut National de Recherche pour l’Agriculture, l’alimentation et l’Environnement (INRAE), Site Agroparc, Domaine Saint-Paul, CS 40509, 84914 Avignon Cedex 9, France; UR406 Abeilles et Environnement, Institut National de Recherche pour l’Agriculture, l’alimentation et l’Environnement (INRAE), Site Agroparc, Domaine Saint-Paul, CS 40509, 84914 Avignon Cedex 9, France; UR406 Abeilles et Environnement, Institut National de Recherche pour l’Agriculture, l’alimentation et l’Environnement (INRAE), Site Agroparc, Domaine Saint-Paul, CS 40509, 84914 Avignon Cedex 9, France; UR406 Abeilles et Environnement, Institut National de Recherche pour l’Agriculture, l’alimentation et l’Environnement (INRAE), Site Agroparc, Domaine Saint-Paul, CS 40509, 84914 Avignon Cedex 9, France; UR406 Abeilles et Environnement, Institut National de Recherche pour l’Agriculture, l’alimentation et l’Environnement (INRAE), Site Agroparc, Domaine Saint-Paul, CS 40509, 84914 Avignon Cedex 9, France; UR406 Abeilles et Environnement, Institut National de Recherche pour l’Agriculture, l’alimentation et l’Environnement (INRAE), Site Agroparc, Domaine Saint-Paul, CS 40509, 84914 Avignon Cedex 9, France

**Keywords:** cantaloupe melon, honey bee colony, pollen supplementation, pollinating activity, stigmatic pollen load

## Abstract

The western honey bee (*Apis mellifera* L.) is the most globally used managed pollinator species, but it can have limited pollinating activity on nectariferous crops displaying anthers isolated from stigmas, i.e., when anthers are spatially or temporally separated from stigma within or between flowers. We supplemented honey bee colonies with pollen in the combs or in paste form laid on top of the hive frames to test if these treatments could reduce their pollen foraging and increase their pollinating activity in a monoecious and nectariferous cultivar of cantaloupe melon (*Cucumis melo* L.), in comparison with control colonies not supplemented. We recorded the pollen forager density per flower, the number of pollen grains deposited per stigma and their resulting fruit set, seed set and fruit mass, before and after the colony pollen supplementations. The number of pollen grains deposited by honey bees on stigmas increased gradually after pollen supplementation in the combs. But pollen foraging decreased only moderately, and no effect could be observed on any yield component except the seed set. On the other hand, there was no effect of the pollen paste laid on top of the frames either on stigmatic pollen loads, on colony pollen foraging or on any yield component. Supplementing honey bee colonies with pollen in the combs can therefore be an effective means for increasing their pollinating activity in nectariferous crops displaying anthers isolated from stigmas, e.g., Amaryllidaceae, Apiaceae, Cucurbitaceae, avocado, all hybrid seed productions. The context for the potential use of pollen substitutes is discussed.

## Introduction

Many crops need insect pollinators to set seeds and fruits (Klein et al. 2007), and managed pollinators are often used in addition to the wild entomofauna to fulfill this need (Isaacs et al. 2017). The western honey bee (*Apis mellifera* L. [Hymenoptera: Apidae]; hereafter ‘honey bee’), is the most globally used managed pollinator species, both in fields and under enclosures. It is often the most effective crop pollinator due to its abundance, however, it is not the most efficient per flower visit in many cases (Rader et al. 2009, Garibaldi et al. 2013, Hung et al. 2018, Page et al. 2021, Junqueira et al. 2022). To increase the pollinating activity of honey bees in crops, growers usually try to increase the honey bee density by increasing the colony stocking rate, while this practice does not necessarily result in better yields and can even be detrimental (Aizen et al. 2014, 2020, Gaines-Day and Gratton 2016, Rollin and Garibaldi 2019).

The limited pollination performance of honey bees can be explained by several behavioral traits. First, honey bees can have limited single-visit pollen depositions (Hung et al. 2018, Földesi et al. 2021, Page et al. 2021). This behavioral trait may result at least partly from their intermediate body size and limited hairiness (Stavert et al. 2016, Roquer-Beni et al. 2020, Földesi et al. 2021). Honey bees can also have poor flower handling behavior for pollination such as nectar theft, a behavior in which they visit flowers from the side to get access to the nectar without contacting the reproductive structures (e.g., Dedej and Delaplane 2005, Park et al. 2016). The pollen carried by pollen foragers in their corbiculae or on their bodies has poor viability compared to the pollen carried by nectar foragers on their bodies or by noncorbiculate bees in their scopae, as honey bee pollen foragers moisten the pollen with oral secretions that affect its viability when grooming (Vaissière et al. 1996, Parker et al. 2015). Honey bees are not particularly effective in transferring cross-pollen on cultivars requiring cross-pollination, especially when wild entomofauna is absent (Jackson 1996, Brittain et al. 2013, Garibaldi et al. 2013, Eeraerts et al. 2020). Finally, honey bees forage in a large area around their nest and they communicate to each other the locations of the most profitable flower patches with dances, resulting in a high ability to be diluted around the target crop on attractive competing blooms (Jay 1986, Osterman et al. 2021, Farina et al. 2023).

To mitigate these behavioral traits of honey bees for pollination, various management methods have been proposed to improve their pollinating activity. For instance, to recruit and direct foragers towards the target crop, beekeepers can feed colonies with caffeine- or arginine-treated sugar syrup scented with the crop floral odors before the onset of flowering (Farina et al. 2023), or they can introduce colonies sequentially into the crop to offset the gradual increase of foraging range that occurs after colony relocation (Al-Tikrity et al. 1972, Gary et al. 1978, Mayer 1994, Shafir 2011). In hermaphrodite crops that experience nectar theft from nectar foragers and for which pollen foragers are better pollinators, like apple tree (*Malus domestica* Borkh. [Rosales: Rosaceae]; Robinson and Fell 1981, Thomson and Goodell 2001), or in crops that rely only on pollen foragers for pollination such as kiwifruit (*Actinidia deliciosa* C. F. Liang & A. R. Ferguson C. F. Liang & A. R. Ferguson [Ericales: Actinidiaceae]), some authors have proposed to increase the pollen foraging activity by fitting hives with pollen traps (Webster et al. 1985, Gemeda et al. 2018), by treating them with brood pheromone (Pankiw 2004, Sagili et al. 2015), or by removing pollen stores (Tsirakoglou et al. 1997). The sequential introduction of colonies and the provision of bumble bee colonies or mason bees can also help decreasing honey bee nectar theft and enhancing cross-pollination by increasing honey bee movements between rows of different cultivars (Stern et al. 2001, 2004, 2005, Brittain et al. 2013, Sapir et al. 2017, Eeraerts et al. 2020). Beekeepers can also feed their colonies with unscented sugar syrup, pollen paste, or pollen substitutes in late winter and during crop flowering to stimulate earlier egg-laying by the queen and thus benefit from more populous colonies, with more sealed and unsealed brood, more foragers including pollen foragers when crops bloom in spring and summer (Goodwin 1997, Kalev et al. 2002, Geslin et al. 2017a, Cavigliasso et al. 2021, Noordyke and Ellis 2021, Hoover et al. 2022). But supplementing colonies with pollen was never contemplated as a means to directly control their level of pollen foraging activity, regardless of colony and unsealed brood size.

Pollen foraging can be responsible for pollen theft, a phenomenon that occurs especially when anthers are isolated from stigmas in nectar-secreting flowers, and especially with honey bees which have been documented as pollen thieves much more frequently than other species (Ish-Am and Eisikowitch, 1993, Young et al. 2007, Hargreaves et al. 2010, 2012, Koski et al. 2018; review in Hargreaves et al. 2009). Isolation of anthers from stigmas occurs when anthers are spatially or temporally separated from stigmas within or between flowers. It appears in a wide range of nectariferous (i.e., with nectar-secreting flowers) entomophilous crop species, listed in Supplementary Table S1 (62 crop species listed, with definitions of the following botanic words): monoecious (e.g., Cucurbitaceae), (gyno)dioecious (e.g., all hybrid seed crops), (hetero/duo)dichogamous (e.g., avocado, *Persea americana* Mill. Mill. [Laurales: Lauraceae]; Amaryllidaceae, Apiaceae, and Asteraceae seed crops), and herkogamous crop species (e.g., strawberry, *Fragaria* × *ananassa* Duchesne Duchesne [Rosales: Rosaceae]).

When anthers are isolated from stigmas in nectariferous flower species, pollination is mainly realized by nectar foragers which visit both pistillate and staminate flowers for nectar, while pollen foragers act mainly as pollen thieves by focusing on staminate flowers and removing pollen from the anthers and the plant population without contacting or pollinating stigmas (Ish-Am and Eisikowitch, 1993, Young et al. 2007). In return, this pollen is lost to the plant population while it could have been used by nectar foragers for pollination (Hargreaves et al. 2009, 2010, Koski et al. 2018). In other words, the activity of pollen foragers in such species reduces the pollinating activity of nectar foragers, through a decrease of the pollen available on the body of nectar foragers.

Nectar and pollen foraging are genetically segregated behaviors among honey bee foragers, and pollen foraging has the distinctive feature of being plastic, depending both on the colony pollen needs on one hand, determined by the pollen consumption of adult bees and larvae, and on its pollen stores on the other hand (Fewell and Winston 1992, Camazine 1993, Fewell and Page 1993, 2000, Page et al. 1995, Pankiw et al. 1998, Dreller et al. 1999, Vaughan and Calderone 2002, Pankiw 2004). Pollen foraging is thus regulated around a certain homeostatic set point between pollen consumption and pollen stores (Fewell and Winston 1992, Camazine 1993, Pankiw et al. 1998, Fewell and Bertram 1999, Fewell and Page 2000, Schmickl and Crailsheim 2004), unlike nectar foraging which is activated without limit as long as there is empty space still available in combs to unload nectar (Fewell and Winston 1996). Seeley (1995) assessed that a honey bee colony maintains a sufficient pollen reserve to meet its needs for about 10 days.

Conceptually, when pollen stores are below this set point, the colony pollen foraging is activated, the activation being mostly achieved by the increase in the number of bees foraging for pollen, and, to a lesser extent, through an increased individual foraging effort of the bees already involved in pollen collection. This can take place through the increase in the number of trips achieved per day, the time spent outside the nest, and the size of the pollen loads they return with to the nest (Fewell and Winston 1992, Fewell and Page 1993, 2000, Pankiw et al. 1998, Fewell and Bertram 1999, Janmaat et al. 2000, Rotjan et al. 2002, Weidenmüller and Tautz 2002, Pankiw 2004). Conversely, when pollen stores are above this set point, pollen foraging is inhibited, with only a few foragers still actively collecting pollen and with a decreased individual foraging effort (Camazine 1993, Fewell and Bertram 1999, Fewell and Page 2000, Janmaat et al. 2000, Weidenmüller and Tautz 2002). When pollen foraging is activated, new pollen foragers are recruited mostly from previously nonforager and nest-related tasks-occupied bees, and, to a lesser extent, among previously nectar foragers (Fewell and Bertram 1999, Rotjan et al. 2002; but see Fewell and Page 1993). Conversely, when pollen foraging is inhibited, bees that stop foraging for pollen mostly just stop foraging altogether and stay in the nest, and only a few of them switch to nectar foraging (Camazine 1993). Consequently, we can expect that supplementing a honey bee colony with pollen in its combs close to unsealed brood can lead to a decrease in its pollen foraging and pollen theft in nectariferous crops displaying anthers isolated from stigmas, and thereby increase the pollinating activity of its nectar foragers through the increased availability of pollen in the crop and on their body.

Cantaloupe melon (*Cucumis melo* L. [Cucurbitales: Cucurbitaceae]; hereafter ‘melon’) is a self-compatible and self-fertile crop which relies entirely on insects for pollination and fruit production, and growers use honey bees worldwide to ensure adequate pollination and getting marketable fruits (Mann 1953, Bohn and Davis 1964, Norden 1985, Kouonon et al. 2009, Delaplane 2023). Grown in open fields or in enclosures (e.g., Dag and Eisikowitch 1995, Rodrigo Gomez et al. 2021), melon production continuously increased during the last 60 yr, along with the global area harvested and yield, to reach 28.6 million tons in the world in 2021, with China accounting for 49% of this total (Supplementary Fig. S1; FAOSTAT 2023). It is also a crop that is strongly resource limited and sets into fruits only a small fraction of its pistillate flowers, even when these flowers are well-pollinated (Mann and Robinson 1950, Mann 1953, Norden 1985, Valantin et al. 1999, Valantin-Morison et al. 2006, Pitrat 2008). Flowers last only 1 day, with anthesis occurring in the morning (Norden 1985, Revanasidda and Belavadi 2019). Most cultivars are andromonoecious, but some are monoecious (Pitrat 2008, 2013). Both pistillate and staminate flowers secrete nectar from anthesis until early afternoon (Orr and Eisikowitch 1988, Dag and Eisikowitch 1999), pollen is also released over this period in staminate flowers (Mann 1953, Orr and Eisikowitch 1988), and the stigma is receptive during this period as well in pistillate flowers (Norden 1985). Consequently, bee foraging and pollination occur mostly during this period (Grewal and Sidhu 1983, Dag and Eisikowitch 1995, 1999, Vaissière and Froissart 1996, Tschoeke et al. 2015). Honey bees forage in melon flowers for both pollen and nectar (Mann 1953, Vaissière et al. 1996). Pistillate flowers require a minimum of 10–15 bee visits per flower to maximize fruit set and fruit size (McGregor et al. 1965, Grewal and Sidhu 1983, Revanasidda and Belavadi 2019).

The aim of our study was to test if supplementing combs of honey bee colonies with pollen could increase their pollinating activity in a nectariferous crop with anthers isolated from stigmas. We used a monoecious cultivar of cantaloupe melon grown in enclosures, to ensure that the crop was pollinated by the treated colonies, and only by them. To assess the colony pollinating activity, we counted the number of melon pollen grains deposited onto stigmas. The resulting fruit set, seed set, and fruit mass were also recorded, although we did not necessarily expect an effect of supplementing honey bee colonies with pollen on these variables since melon production is strongly resource limited. Despite its strong resource limitation, melon crop has the advantage of having flowers that last only 1 day, which enabled us to have multiple independent repetitions by sampling the stigmatic pollen loads over several independent days. We also had a method to count the pollen grains on dry stigmas for this species (Vaissière and Froissart 1996), enabling us to use melon as a model for proof of concept for our hypothesis. In addition, we also supplemented colonies with pollen in paste form laid on top of the hive frames, as it is commonly used to increase the colony and brood size, and sometimes in crop pollination contexts (Kalev et al. 2002, Noordyke and Ellis 2021, Hoover et al. 2022), to test if it might provide an easier means to decrease the colony pollen foraging and increase their pollinating activity.

## Materials and Methods

### Study Sites and Crop Model

This study was carried out from April to June 2011 in 6 commercial tunnels located in Avignon in a commercial farm (France; 43°54ʹ46.12″N, 4°52ʹ35.64″E). The vents of the 71 m long × 9.4 m wide tunnels were covered with insectproof polyethylene crystal screen with 8 mm × 3 mm openings (F1028Q; Diatex, France) to prevent honey bees from leaving the tunnels. Each tunnel was sown with 4 rows of the monoecious cantaloupe melon cv. F_1_ hybrid “Neo” (*Cucumis melo* L. ssp. *melo* var. *cantalupensis* horticultural group “Charentais”; Supplementary Fig. S2).

### Experimental Setup, and Counts of Flowers and Honey Bee Foragers

The experiment was organized in 2 repetitions of 3 tunnels each, the samplings occurring every 2 days from 28 April to 12 May 2011 for the first repetition, and from 13 May to 27 May 2011 for the second repetition, with 8 sampling days for each repetition. One small honey bee colony in a 5-frame Dadant hive supplied from a standard apiary was introduced in each tunnel, as usually practiced on the farm, at 13:00 UTC (3 PM) 2 days before the beginning of the samplings. A different colony was used for each repetition. The pistillate and staminate flowers were counted on each sampling day at ca. 12:00 UTC (2 PM) in each tunnel in 4 plots 2 m long distributed around the geometric center of each tunnel and located at least 5 m apart from the tunnel edges (see Supplementary Fig. S3), each plot being in a different row. Honey bee foragers were counted twice on each sampling day at ca. 08:00 UTC (10 AM) and 12:00 UTC (2 PM) on 200 flowers randomly chosen each time in each tunnel. Each flower was scanned once by recording if a bee was foraging in it or not at the time of observation (instantaneous count; Vaissière et al. 2011, Garibaldi et al. 2016). The same flowers could be observed at 08:00 UTC (10 AM) and then again at 12:00 UTC (2 PM) depending on the random choice. The 200 flowers were divided into 4 transects, with 1 transect of 50 flowers per row. Each transect started from the plot of flower count and headed towards the geometric center of each tunnel (Vaissière et al. 2011; see Supplementary Fig. S3). The scanned flowers were either pistillate or staminate at random. For each forager scanned, we recorded the presence or absence of pollen in its corbiculae.

### Assessment of Honey Bee Colonies and Pollen Supplementation Treatments

In the evening of the 5th sampling day of each repetition, i.e., 6 May for repetition 1 and 21 May for repetition 2, each honey bee colony was assessed at night. The frames, the hive, and the bottom board of each colony were shaken and brushed above an empty swarm box at night with the aim to catch all the adult bees of the colony in the box. This box was weighed, and its empty mass subtracted to get the mass of the bee population. When the bees were returned to their hive, a sample of ca. 100 bees was taken from the population, frozen, weighed, and counted later to obtain the mean mass per bee. This mean mass was used to convert the mass of the bee population into a number of bees (Farrar 1937, Chabert et al. 2021). The areas of unsealed and sealed brood, and of pollen and honey stores were assessed for each colony by placing a clear acrylic plexiglass sheet on each frame side and by drawing the outline of each component with a marker. The drawn areas were then analyzed with the plugin *Versatile Wand Tool* (https://imagej.nih.gov/ij/plugins/versatile-wand-tool/index.html) of the software ImageJ (Schneider et al. 2012). The colony features are given in Supplementary Table S2. These features enabled us to calculate that the amount of 1 kg of pollen used for the pollen supplementation treatments (see below) was large enough to exceed the homeostatic set point beyond which the colonies decrease their pollen collection: 1 kg of pollen was equivalent to more than 3 wk of pollen consumption of the colony presenting the highest daily pollen consumption, while a honey bee colony usually maintains a sufficient pollen reserve to meet its needs for about 10 days only (Seeley 1995; see Supplementary material, Section 2 for the calculations).

**Fig. 1.**
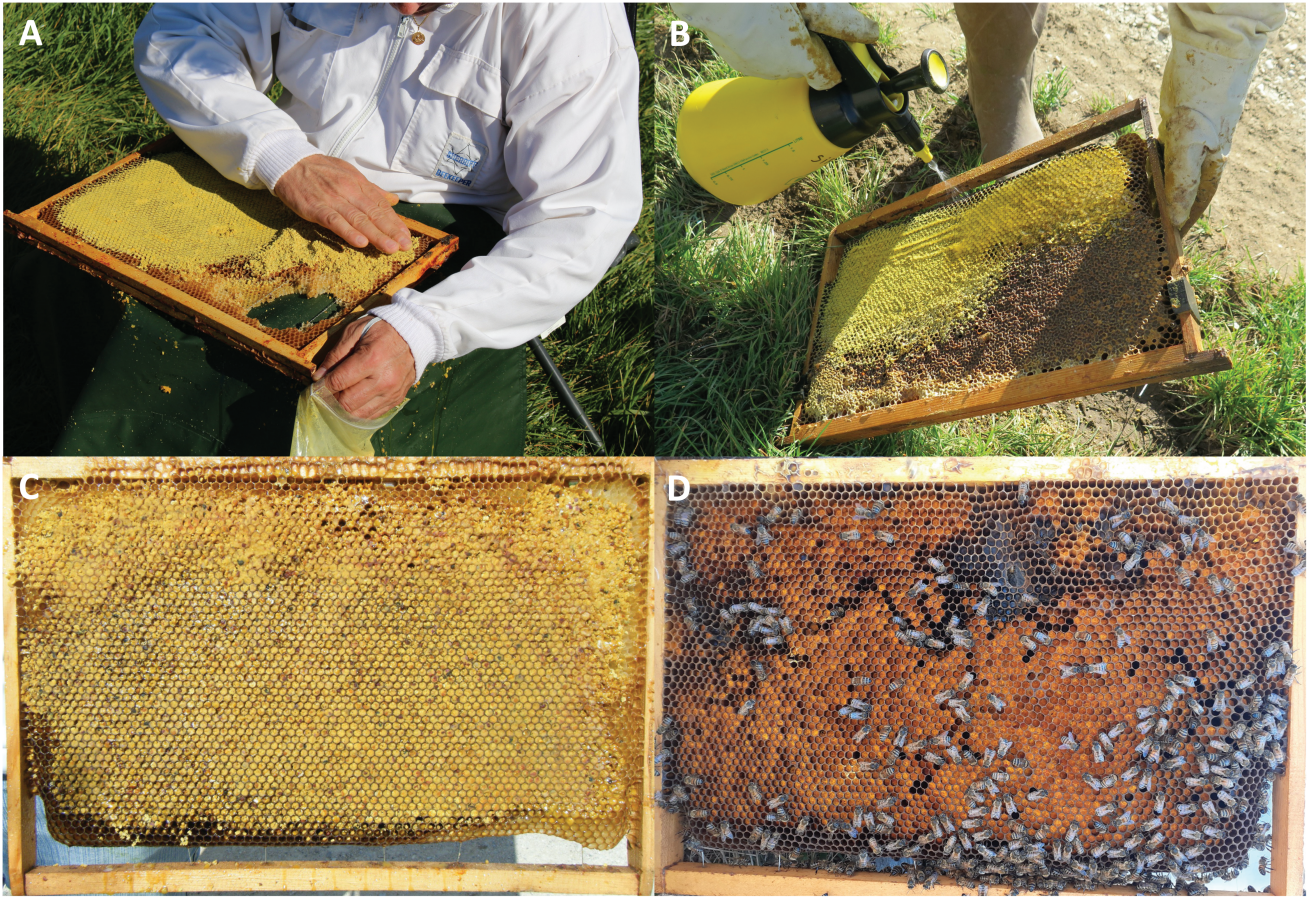
(A) Insertion of thawed bee pollen pellets in the combs. (B) Spraying of saccharose syrup on the combs. (C) The pollen pellets sprayed with syrup remain in the combs when the frame is held vertically. (D) All the pollen pellets have been packed into bee bread and partly consumed by the honey bees one week after. Photo credit: Cyril Scomparin/INRAE and Stan Chabert/INRAE.

**Fig. 2.**
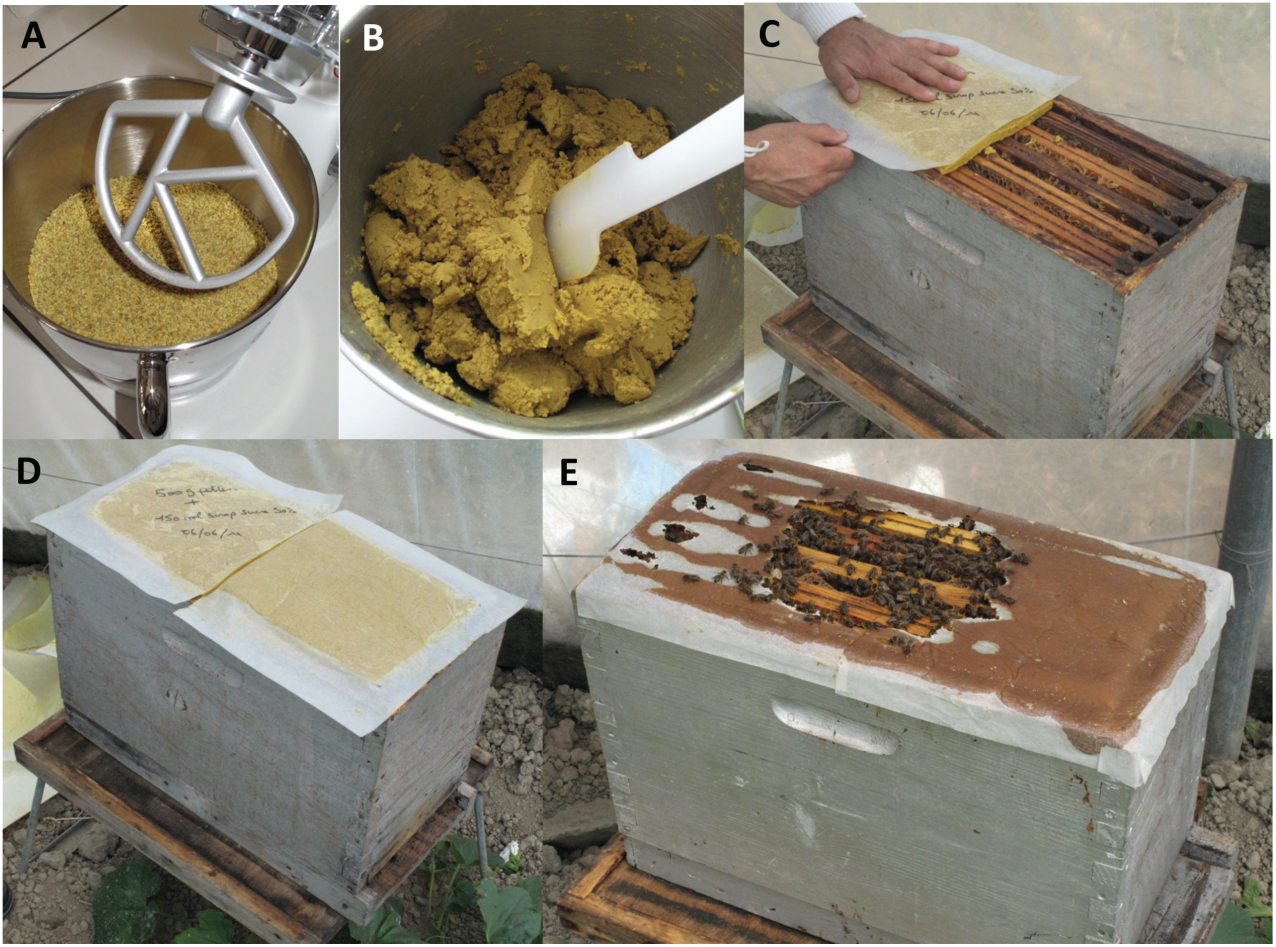
(A, B) Mixing of bee pollen pellets with saccharose syrup to prepare the pollen paste. (C, D) Deposition of the pollen paste on top of the hive frames. (E) The pollen paste has been partly consumed by the honey bees one week after. Photo credit: Laurent Guilbaud/INRAE and Nicolas Morison/INRAE.

Following this assessment, each colony out of the 3 within a repetition was treated randomly as follows: 1 colony did not receive any pollen supplementation (= control), 1 colony received a supplementation of 1 kg of pollen in the form of bee pollen pellets inserted in the combs (Fig. 1; Supplementary Material, Section 2), and 1 colony received a supplementation of 1 kg of pollen in the form of a paste laid on top of the hive frames (Fig. 2; Supplementary Material, Section 2). Therefore, there were 5 sampling days before colony treatment for the 2 repetitions (28 and 30 April, 2, 4, and 6 May for repetition 1; 13, 15, 17, 19, and 21 May for repetition 2), and 3 sampling days after colony treatment (8, 10, and 12 May for repetition 1; 23, 25, and 27 May for repetition 2).

### Analysis of the Stigmatic Pollen Loads and Recording of the Resulting Yields

On each sampling day, 14 pistillate flowers were randomly chosen per tunnel in plots evenly distributed in each row. To have a complete uniform distribution of samplings, each plot was sampled every 2 sampling days (see Supplementary Fig. S4). Thus, every 2 sampling days, each plot was sampled once, with 1 pistillate flower sampled on 1 plant. Each time a plot was sampled, a different plant was used. The flowers were labeled with the date and their location identified with a small flag. The stigmas were collected 10 days later not to interfere with the fertilization process. All the pollen grains stay on the stigma during this time interval due to a high adhesion force (Ito and Gorb 2019). These stigmas were individually stored in 1.5 ml plastic tubes and dried for 24 h at 30 °C in a ventilated incubator. To accurately count the number of melon pollen grains deposited on the stigmas, the pollen grains were extracted by sonication in a solution of saline water and malachite green (Vaissière 1991, Vaissière and Froissart 1996), and the resulting pollen suspensions were filtered on white nylon membrane (see Supplementary Material, Section 3). The pollen grains stained with malachite green were then counted by image analysis with the software ImageJ (see Supplementary Material, Section 3).

To relate the stigmatic pollen loads to the resulting yields, each fruit produced from a flower from which the stigma was sampled was harvested at maturity, weighed and dissected to remove the seeds. The filled seeds were separated from the empty hulls, before being dried and counted for each fruit with a seed counter (Contador; Pfeuffer, Germany). The fruit set, i.e., the % of pistillate flowers setting a fruit, was also recorded.

### Data Analyses

We tested first if there was a difference in the numbers of pistillate and staminate flowers between the 3 colony treatments, as it could have interfered with the effects of the colony treatments on pollination. The numbers of pistillate and staminate flowers counted on each sampling day in the 4 rows of each tunnel were analyzed by grouping the 2 repetitions with generalized linear mixed effect models (GLMMs) with Poisson regression, after checking that there was no overdispersion (i.e., with the dispersion parameter *Φ* close to 1; Zuur et al. 2009). The % of staminate flowers per tunnel and per day was analyzed with a GLMM with binomial regression. For each model, the colony treatment (control colony, colony supplemented with pollen pellets in the combs, or colony supplemented with pollen paste) and the experimental period (before or after colony treatment) were set as fixed explanatory variables with an interaction term, and the tunnel and sampling date were set as random effects.

To test if the colony treatment impacted pollen foraging, the number of pollen foragers counted on 200 flowers each day in each tunnel was analyzed by grouping the 2 repetitions with a GLMM with Poisson regression, after checking the absence of overdispersion as well. The % of pollen foragers per tunnel per day was analyzed with a GLMM with binomial regression (success: number of foragers per 200 flowers with pollen in their corbiculae; failure: number of foragers per 200 flowers without pollen in their corbiculae). As before, the colony treatment and the experimental period were set as fixed explanatory variables with an interaction term, and the tunnel and sampling dates were set as random effects.

To test if the colony treatment impacted the pollination of pistillate flowers, the number of pollen grains per stigma was analyzed by grouping the 2 repetitions with a GLMM, using the quadratic negative binomial regression (NB2; Hilbe 2014) for the residual distribution, because the number of pollen grains per stigma is a nonindependent count (pollen grains are grouped within stigmas) implying overdispersion. As before, the colony treatment and the experimental period were set as fixed explanatory variables with an interaction term, while the tunnel and sampling date were set as random effects.

In addition, to test a potential effect of temporal dynamics of colony treatment on the pollen foraging and pollination activities of honey bees after treatment, the same models described above were run again but by individually comparing each of the 3 sampling dates after treatment (2, 4, and 6 days after treatment) with the average of the 5 sampling dates before treatment.

Finally, to test if the colony treatment impacted melon production, the fruit set was analyzed with a GLMM with binomial regression. The number of filled seeds per fruit being a nonindependent count (seeds are grouped within fruits) implying overdispersion, it was analyzed with a GLMM with NB2 regression. Fruit mass was analyzed with a linear mixed effect model (LMM). As before, the colony treatment and the experimental period were set as fixed explanatory variables with an interaction term, while the tunnel and sampling date were set as random effects.

Since the initial conditions could not necessarily be the same between the tunnels and the colonies between treatments, we applied a Before-After-Control-Impact (BACI) design (Christie et al. 2019, 2020): the impact of the colony treatments on each dependent variable was analyzed by comparing the variations over time before/after treatment of the dependent variables of the treated colonies with the variations of the control colonies through the interaction terms. BACI designs are robust, even when the initial conditions are not the same between the control and treated sites (Christie et al. 2019). To address the issue of a potential high temporal variability of bee foraging and pollinating activity between days, samplings were repeated simultaneously over several days before and after treatment (5 days before treatment and 3 days after): in this sense, this design was a BACI Paired-Series design (BACIPS; Christie et al. 2019). BACI designs are quite robust to detect the true direction of an effect, even when the number of repetitions is low, such as in our study which included 2 repetitions (Christie et al. 2019). In addition, 645 stigmas were analyzed in total over the 2 repetitions and the 3 colony treatments.

The statistics were computed with the software R, version 4.0.3 (R Core Team 2020). The mixed-effects models were computed with the package *lme4*, version 1.1-31 (Bates et al. 2015). The *P*-values of the LMMs were obtained with the package *lmerTest*, version 3.1-3 (Kuznetsova et al. 2017). The NB2 regression was used with the package *MASS*, version 7.3-58.2 (Venables and Ripley 2002). The gradual language of evidence was chosen to describe the results, following the recommendations of Muff et al. (2022): weak statistical evidence when 0.05 ≤ *P* < 0.1, moderate when 0.01 ≤ *P* < 0.05, strong when 0.001 ≤ *P* < 0.01, and very strong when *P* < 0.001. All the ± errors given around the means in the result section are standard errors.

## Results

Six days after pollen supplementations, all the pollen pellets added into the combs had been packed into bee bread and partly consumed (Fig. 1D), and the pollen paste laid on top of the hive frames had been partly consumed as well (Fig. 2E).

### Flower Density

**Fig. 3.**
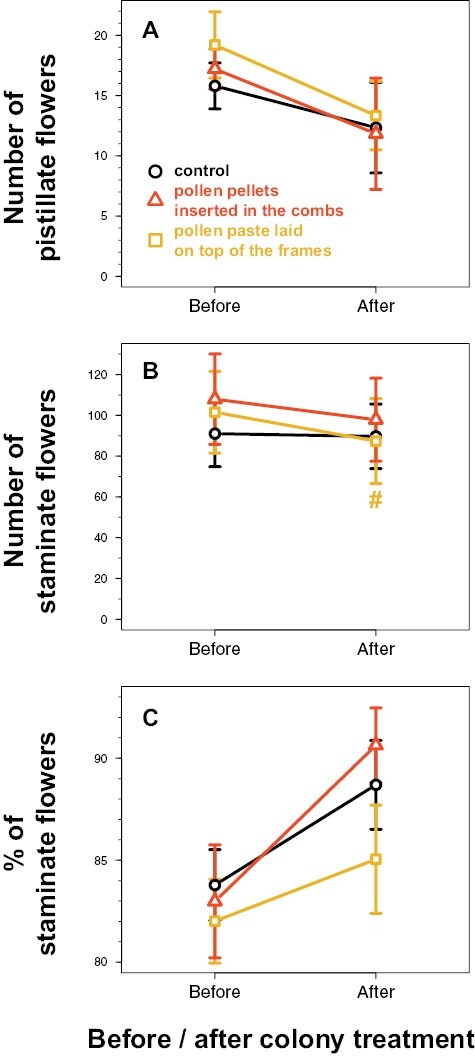
Variation of the flower number (mean ± SE) assessed in 4 plots 2 m long per tunnel per sampling day before (5 sampling days) and after (3 sampling days) colony treatment. The control refers to the tunnels provided with colonies not supplemented with pollen. Each treatment was replicated twice over 2 different periods in independent tunnels. #: indicates weak statistical evidence of the interaction between the colony treatment (control versus pollen supplementation) and the period before/after treatment, i.e., with 0.05 ≤ *P* < 0.1 (Table 1).

Over the 2 repetitions, there was no evidence that the number of pistillate and staminate flowers, as well as the % of staminate flowers, per 8 m transect per tunnel per sampling day varied differently before and after colony treatment, for the tunnels supplemented with pollen compared to the control tunnel (*P* > 0.3 for the interactions; Table 1 and Fig. 3). There was only weak evidence that the number of staminate flowers decreased after treatment in the tunnels supplemented with pollen paste (interaction: *z* = −1.7, *P* = 0.09; Fig. 3B) compared to the control tunnels for which there was no evidence of decrease (*z* = −1.3, *P* = 0.19).

**Table 1.** Statistics of the GLMMs computed to test the differences of flower number, assessed in 8 m transects per tunnel per sampling day, between the 3 colony treatments

Response variable	Predictor	Estimate ± SE	*z*	*P*	Statistical evidence of interactions
Number of pistillate flowers per 8 m transect	Intercept: control - BT	2.705 ± 0.169	17.02	<0.001	
	Control - AT	−0.348 ± 0.267	−1.30	0.193	
	Pollen in the combs - BT	0.085 ± 0.110	0.77	0.439	
	Pollen paste on top of the frames - BT	0.195 ± 0.107	1.82	0.069	
	Interaction: pollen in the combs - AT	−0.126 ± 0.198	−0.64	0.525	
	Interaction: pollen paste - AT	−0.117 ± 0.193	−0.61	0.544	
Number of staminate flowers per 8 m transect	Intercept: control - BT	4.369 ± 0.395	11.05	<0.001	
	Control - AT	0.032 ± 0.088	0.36	0.716	
	Pollen in the combs - BT	0.117 ± 0.556	0.21	0.833	
	Pollen paste on top of the frames - BT	0.052 ± 0.556	0.09	0.925	
	Interaction: pollen in the combs - AT	−0.076 ± 0.075	−1.02	0.308	
	Interaction: pollen paste - AT	−0.128 ± 0.077	−1.68	0.094	Weak
% of staminate flowers	Intercept: control - BT	1.659 ± 0.134	12.41	<0.001	
	Control - AT	0.384 ± 0.227	1.70	0.090	
	Pollen in the combs - BT	0.074 ± 0.120	0.62	0.535	
	Pollen paste on top of the frames - BT	−0.107 ± 0.117	−0.92	0.360	
	Interaction: pollen in the combs - AT	0.053 ± 0.214	0.25	0.802	
	Interaction: pollen paste - AT	−0.003 ± 0.209	−0.01	0.989	

AT, after treatment; BT, before treatment. The rows highlighted in gray correspond to the interaction terms, on which the interpretations are focused to test the hypotheses. Statistical evidence follows recommendations made by Muff et al. (2022).

### Density of Honey Bee Pollen Foragers

**Fig. 4.**
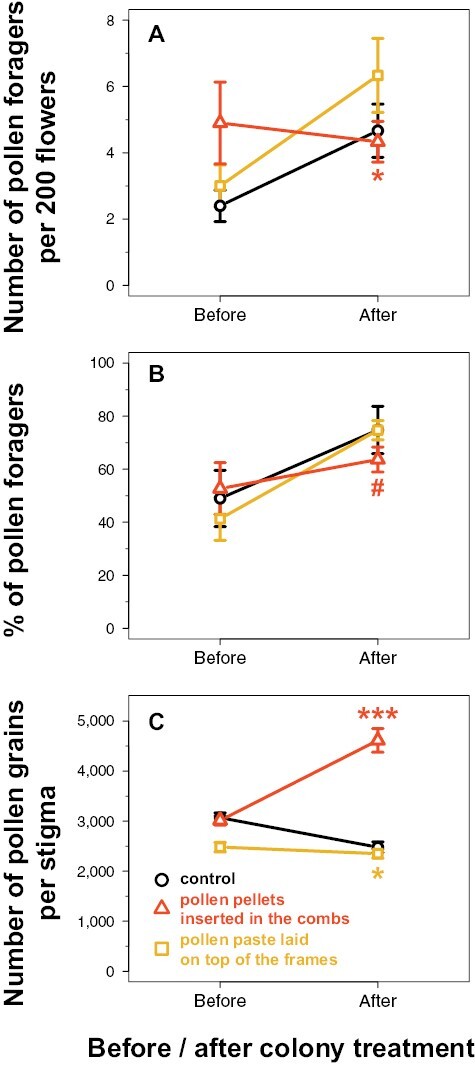
Variation of the honey bee pollen foraging activity (A, B) assessed around 08:00 UTC (10 AM), and of the honey bee pollinating activity (C), assessed before (5 sampling days) and after (3 sampling days) colony treatment (mean ± SE). The control refers to the tunnels provided with colonies not supplemented with pollen. Each treatment was replicated twice over 2 different periods in independent tunnels. Symbols indicate the level of statistical evidence of the interaction between the colony treatment (control versus pollen supplementation) and the period before/after treatment: very strong for *** (*P* < 0.001), moderate for * (0.01 ≤ *P* < 0.05), weak for # (0.05 ≤ *P* < 0.1) (Table 2).

**Fig. 5.**
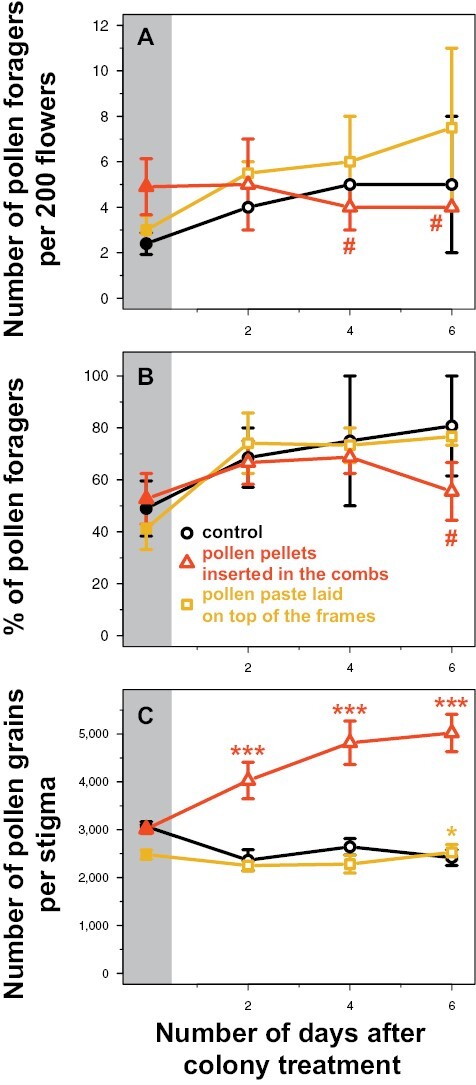
Variation (mean ± SE) over time after colony treatment of the honey bee pollen foraging activity (A, B) assessed around 08:00 UTC (10 AM), and of the honey bee pollinating activity (C). The control refers to the tunnels provided with colonies not supplemented with pollen. Each treatment was replicated twice over 2 different periods in independent tunnels. The gray areas correspond to the averages of the 5 dates sampled before treatment across the 2 tunnels for each treatment. Symbols indicate the level of statistical evidence of the interaction between the colony treatment (control versus pollen supplementation) and the period before/after treatment: very strong for *** (*P* < 0.001), moderate for * (0.01 ≤ *P* < 0.05), weak for # (0.05 ≤ *P* < 0.1) (Table 3).

As honey bees foraged for pollen almost exclusively in the morning (Supplementary Fig. S5), the analyses of pollen foragers focused on the counts made around 08:00 UTC (10 AM). Over the 2 repetitions, the number of pollen foragers decreased with moderate evidence in the tunnels supplemented with pollen in the combs (interaction: *z* = −2.2, *P* = 0.03; Table 2 and Fig. 4A), from 4.9 ± 1.2 pollen foragers per 200 flowers on average before treatment to 4.3 ± 0.6 after treatment, compared to the control tunnels in which this number increased with moderate evidence (*z* = 2.1, *P* = 0.03), from 2.4 ± 0.5 pollen foragers before treatment to 4.7 ± 0.8 after treatment. More precisely, the number of pollen foragers decreased with weak evidence to 4.0 (± 1.0 and 0.0, respectively) pollen foragers per 200 flowers in average on the fourth and sixth days after treatment in the tunnels supplemented with pollen in the combs (interactions: *z* = −1.8, *P* = 0.08; Table 3 and Fig. 5A), while it increased with weak evidence to 5.0 (± 0.0 and 3.0, respectively) pollen foragers during the same period in the control tunnels (*z* = 1.7, *P* = 0.09-0.10). There was no evidence that the number of pollen foragers varied differently on the 2^nd^ day after treatment in the tunnels supplemented with pollen in the combs compared to the control tunnels (interaction: *z* = −0.9, *P* = 0.36). On the other hand, there was no evidence that the number of pollen foragers varied differently before and after colony treatment for the tunnels supplemented with pollen paste compared to the control tunnels (*P* > 0.7 for the interactions; Tables 2 and 3, Figs. 4A and 5A).

**Table 2.** Statistics of the GLMMs computed to test the effect of colony treatment on honey bee pollen foraging and pollinating activity

Response variable	Predictor	Estimate ± SE	*z*	*P*	Statistical evidence of interactions
Number of pollen foragers per 200 flowers	Intercept: control - BT	0.781 ± 0.243	3.22	0.001	
	Control - AT	0.732 ± 0.344	2.13	0.033	
	Pollen in the combs - BT	0.714 ± 0.247	2.89	0.004	
	Pollen paste on top of the frames - BT	0.223 ± 0.272	0.82	0.411	
	Interaction: pollen in the combs - AT	−0.788 ± 0.366	−2.15	*0.031*	*Moderate*
	Interaction: pollen paste - AT	0.082 ± 0.367	0.22	0.823	
% of pollen foragers	Intercept: control - BT	−0.436 ± 0.301	−1.45	0.148	
	Control - AT	1.209 ± 0.484	2.50	0.013	
	Pollen in the combs - BT	0.805 ± 0.365	2.21	0.027	
	Pollen paste on top of the frames - BT	0.116 ± 0.364	0.32	0.750	
	Interaction: pollen in the combs - AT	−1.080 ± 0.587	−1.84	0.066	Weak
	Interaction: pollen paste - AT	0.207 ± 0.597	0.35	0.729	
Number of pollen grains per stigma	Intercept: control - BT	8.017 ± 0.083	96.26	<0.001	
	Control - AT	−0.205 ± 0.078	−2.62	0.009	
	Pollen in the combs - BT	−0.013 ± 0.107	−0.12	0.904	
	Pollen paste on top of the frames - BT	−0.212 ± 0.107	−1.98	0.048	
	Interaction: pollen in the combs - AT	0.590 ± 0.076	7.77	**<0.001**	**Very strong**
	Interaction: pollen paste - AT	0.163 ± 0.075	2.16	*0.030*	*Moderate*

AT, after treatment; BT, before treatment. The rows highlighted in gray correspond to the interaction terms, on which the interpretations are focused to test the hypotheses. Statistical evidence follows recommendations made by Muff et al. (2022). Moderate evidence (0.01 ≤ *P* < 0.05) is highlighted in italics; very strong evidence (*P* < 0.001) is highlighted in bold.

**Table 3.** Statistics of the GLMMs computed to test the effect of colony treatment on honey bee pollen foraging and pollinating activity, testing independently each sampling date after colony treatment in comparison with the average of the sampling dates before treatment

Response variable	Predictor	Estimate ± SE	*z*	*P*	Statistical evidence of interactions
Number of pollen foragers per 200 flowers	Intercept: control - BT	0.781 ± 0.242	3.22	0.001	
	Control - 2 days AT	0.579 ± 0.504	1.15	0.251	
	Control - 4 days AT	0.796 ± 0.479	1.66	0.097	
	Control - 6 days AT	0.808 ± 0.479	1.69	0.092	
	Pollen in the combs - BT	0.715 ± 0.247	2.89	0.004	
	Pollen paste on top of the frames - BT	0.224 ± 0.272	0.83	0.409	
	Interaction: pollen in the combs - 2 days AT	−0.491 ± 0.532	−0.92	0.355	
	Interaction: pollen in the combs - 4 days AT	−0.938 ± 0.532	−1.76	0.078	Weak
	Interaction: pollen in the combs - 6 days AT	−0.938 ± 0.532	−1.76	0.078	Weak
	Interaction: pollen paste - 2 days AT	0.095 ± 0.535	0.18	0.859	
	Interaction: pollen paste - 4 days AT	−0.042 ± 0.504	−0.08	0.934	
	Interaction: pollen paste - 6 days AT	0.181 ± 0.488	0.37	0.710	
% of pollen foragers	Intercept: control - BT	−0.361 ± 0.367	−0.99	0.324	
	Control - 2 days AT	1.152 ± 0.709	1.62	0.104	
	Control - 4 days AT	1.199 ± 0.652	1.84	0.066	
	Control - 6 days AT	1.318 ± 0.672	1.96	0.050	
	Pollen in the combs - BT	0.782 ± 0.475	1.65	0.100	
	Pollen paste on top of the frames - BT	0.075 ± 0.478	0.16	0.875	
	Interaction: pollen in the combs - 2 days AT	−0.942 ± 0.896	−1.05	0.293	
	Interaction: pollen in the combs - 4 days AT	−0.846 ± 0.911	−0.93	0.353	
	Interaction: pollen in the combs - 6 days AT	−1.567 ± 0.866	−1.81	0.071	Weak
	Interaction: pollen paste - 2 days AT	0.189 ± 0.935	0.20	0.839	
	Interaction: pollen paste - 4 days AT	0.260 ± 0.886	0.29	0.769	
	Interaction: pollen paste - 6 days AT	0.005 ± 0.901	0.01	0.995	
Number of pollen grains per stigma	Intercept: control - BT	8.017 ± 0.083	96.82	< 0.001	
	Control - 2 days AT	−0.254 ± 0.115	−2.21	0.027	
	Control - 4 days AT	−0.137 ± 0.113	−1.22	0.224	
	Control - 6 days AT	−0.233 ± 0.115	−2.02	0.043	
	Pollen in the combs - BT	−0.013 ± 0.107	−0.12	0.903	
	Pollen paste on top of the frames - BT	−0.212 ± 0.107	−1.97	0.049	
	Interaction: pollen in the combs - 2 days AT	0.492 ± 0.113	4.34	**<0.001**	**Very strong**
	Interaction: pollen in the combs - 4 days AT	0.565 ± 0.115	4.93	**<0.001**	**Very strong**
	Interaction: pollen in the combs - 6 days AT	0.719 ± 0.114	6.32	**<0.001**	**Very strong**
	Interaction: pollen paste - 2 days AT	0.164 ± 0.113	1.45	0.146	
	Interaction: pollen paste - 4 days AT	0.071 ± 0.112	0.63	0.527	
	Interaction: pollen paste - 6 days AT	0.261 ± 0.114	2.28	*0.022*	*Moderate*

AT, after treatment; BT, before treatment. The rows highlighted in gray correspond to the interaction terms, on which the interpretations are focused to test the hypotheses. Statistical evidence follows recommendations made by Muff et al. (2022). Moderate evidence (0.01 ≤ *P* < 0.05) is highlighted in italics; very strong evidence (*P* ≤ 0.001) is highlighted in bold.

There was weak evidence that the % of pollen foragers increased less in the tunnels supplemented with pollen in the combs (interaction: *z* = −1.8, *P* = 0.07; Table 2 and Fig. 4B), from 52.7% ± 9.7 on average before treatment to 63.7% ± 4.7 after treatment, compared to the control tunnels in which this % increased with moderate evidence, from 49.0% ± 10.6 before treatment to 74.8% ± 8.9 after treatment (*z* = 2.5, *P* = 0.01). More precisely, there was weak evidence that the % of pollen foragers increased less on the sixth day after treatment in the tunnels supplemented with pollen in the combs compared to the control tunnels (interaction: *z* = −1.8, *P* = 0.07; Table 3 and Fig. 5B): it reached 55.6% ± 11.1 on average in the tunnels supplemented with pollen in the combs on the sixth day after treatment while it reached 80.8% ± 19.2 in the control tunnels (*z* = 2.0, *P* = 0.05). There was no evidence that the % of pollen foragers varied differently on the 2nd and 4th days after treatment in the tunnels supplemented with pollen in the combs compared to the control tunnels (interactions: *P* > 0.2). On the other hand, there was no evidence that the % of pollen foragers varied differently before and after colony treatment for the tunnels supplemented with pollen paste compared to the control tunnels (*P* > 0.7 for the interactions; Tables 2 and 3, Figs. 4B and 5B).

### Stigmatic Pollen Loads

While the stigmatic pollen load decreased in the control tunnels with strong evidence over the 2 repetitions (*z* = −2.6, *P* = 0.009; Table 2 and Fig. 4C), from 3,069 ± 97 pollen grains on average per stigma before treatment to 2,479 ± 107 after treatment, it increased with very strong evidence, from 3,014 ± 94 before treatment to 4,614 ± 237 after treatment, i.e., a 1.5-fold increase, in the tunnel supplemented with pollen in the combs (interaction: *z* = 7.8, *P* < 0.001). More precisely, the stigmatic pollen load increased in the tunnel supplemented with pollen in the combs with very strong evidence to 4,028 ± 382, 4,816 ± 454, and 5,022 ± 390 pollen grains per stigma on the second, fourth, and sixth days after treatment, i.e., a 1.3-, 1.6-, and 1.7-fold increase, respectively, after treatment (*P* < 0.001 for the 3 interactions; Table 3 and Fig. 5C), compared to the control tunnels in which the stigmatic pollen load decreased with moderate evidence on the second and sixth days after treatment (*z* = −2.2, *P* = 0.03, and *z* = −2.0, *P* = 0.04, respectively), and without evidence of variation on the fourth day after treatment (*z* = −1.2, *P* = 0.22).

There was moderate evidence that the stigmatic pollen loads decreased less in the tunnels supplemented with pollen paste (interaction: *z* = 2.2, *P* = 0.03; Table 2 and Fig. 4C), from 2,482 ± 96 on average before treatment to 2,350 ± 90 after treatment, compared to the control tunnels. More precisely, there was moderate evidence of no decrease of the stigmatic pollen loads in the tunnels supplemented with pollen paste on the sixth day after treatment compared to the control tunnels (interaction: *z* = 2.3, *P* = 0.02; Table 3 and Fig. 5C), and no evidence of different variation between the tunnels supplemented with pollen paste and the control tunnel on the second and fourth days after treatment (*P* > 0.1 for the 2 interactions).

### Fruit Set, Seed Set, and Fruit Mass

**Fig. 6.**
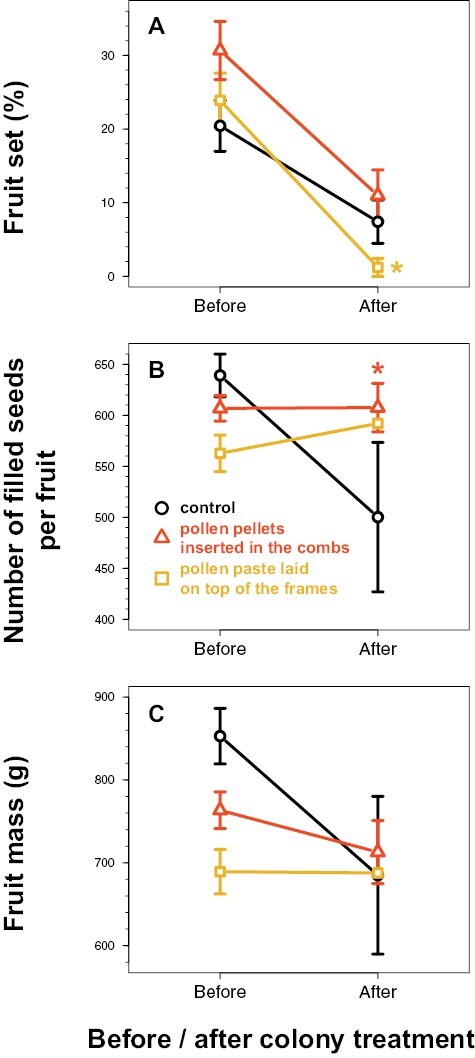
Variation of the yield variables (mean ± SE), assessed before (5 sampling days) and after (3 sampling days) colony treatment. The control refers to the tunnels provided with colonies not supplemented with pollen. Each treatment was replicated twice over 2 different periods in independent tunnels. *: indicates moderate statistical evidence of the interactions between the colony treatment (control versus pollen supplementation) and the period before/after treatment, i.e., with 0.01 ≤ *P* < 0.05 (Table 4).

Over the 2 repetitions, there was no evidence that the fruit set, number of filled seeds per fruit and fruit mass varied differently before and after colony treatment, for the tunnels supplemented with pollen compared to the control tunnel (*P* > 0.1 for the interactions; Table 4 and Fig. 6). There was only moderate evidence that the fruit set decreased after treatment in the tunnels supplemented with pollen paste (interaction: *z* = −2.0, *P* = 0.04; Fig. 6A), from 23.9% ± 3.7 on average before treatment to 1.2% ± 1.2 after treatment, compared to the control tunnels for which there was no evidence of decrease (*z* = −0.9, *P* = 0.35). There was also moderate evidence that the number of filled seeds per fruit did not vary after treatment in the tunnels supplemented with pollen in the combs with an average of 607 ± 11 seeds (interaction: *z* = 2.4, *P* = 0.02; Fig. 6B), compared to the control tunnels for which there was strong evidence of a decrease of the number of filled seeds per fruit (*z* = −3.1, *P* = 0.002), from 639 ± 21 seeds on average before treatment to 500 ± 73 after treatment.

**Table 4.** Statistics of the (G)LMMs computed to test the effect of colony treatment on the 3 yield variables

Response variable	Predictor	Estimate ± SE	*z* or *t*	*P*	Statistical evidence of interactions
Fruit set	Intercept: control - BT	−2.009 ± 0.543	−3.70	<0.001	
	Control - AT	−0.857 ± 0.915	−0.94	0.349	
	Pollen in the combs - BT	0.894 ± 0.361	2.48	0.013	
	Pollen paste on top of the frames - BT	0.344 ± 0.372	0.92	0.356	
	Interaction: pollen in the combs - AT	−0.436 ± 0.651	−0.67	0.504	
	Interaction: pollen paste - AT	−2.245 ± 1.111	−2.02	*0.043*	*Moderate*
Number of filled seeds per fruit	Intercept: control - BT	6.460 ± 0.033	196.12	<0.001	
	Control - AT	−0.245 ± 0.079	−3.11	0.002	
	Pollen in the combs - BT	−0.052 ± 0.043	−1.22	0.223	
	Pollen paste on top of the frames - BT	−0.127 ± 0.045	−2.82	0.005	
	Interaction: pollen in the combs - AT	0.246 ± 0.102	2.42	*0.015*	*Moderate*
	Interaction: pollen paste - AT	0.296 ± 0.194	1.52	0.128	
Fuit mass (g)	Intercept: control - BT	825.8 ± 36.0	22.96	<0.001	
	Control - AT	−141.9 ± 75.4	−1.88	0.065	
	Pollen in the combs - BT	−87.2 ± 36.8	−2.37	0.020	
	Pollen paste on top of the frames - BT	−172.0 ± 39.2	−4.39	<0.001	
	Interaction: pollen in the combs - AT	123.4 ± 87.7	1.41	0.162	
	Interaction: pollen paste - AT	169.2 ± 169.9	1.00	0.322	

AT, after treatment; BT, before treatment. The rows highlighted in gray correspond to the interaction terms, on which the interpretations are focused to test the hypotheses. Statistical evidence follows recommendations made by Muff et al. (2022). Moderate evidence (0.01 ≤ *P* < 0.05) is highlighted in italics.

## Discussion

Given that there was no evidence of different variations before/after treatment for the number of flowers and the ratio of staminate to pistillate flowers between the 3 treatments, the variations in flower numbers most probably did not interfere with the effects of the colony treatments on the pollinating activity of the honey bees. Our results demonstrated that the pollen supplementation in the combs increased by a factor of 1.5 on average over the 2 repetitions of the number of pollen grains deposited on melon stigmas. This increase was gradual after the pollen supplementation: the stigmatic pollen load first increased by 1.3 two days after the supplementation, and then by 1.6 and 1.7 after 4 and 6 days after the supplementation.

These results are in accordance with our hypothesis that supplementing a honey bee colony with pollen in its combs could increase its pollinating activity through the reduction in pollen foraging and pollen theft in a nectariferous crop with anthers isolated from stigmas. However, we observed only weak to moderate evidence of a decrease in the pollen forager density and the % of pollen foragers following the pollen supplementation in the combs compared to the control, a decrease that was rather pronounced 4–6 days after the supplementation. This observation contrasts with several previous studies that showed that increasing the pollen stores of a colony divided by 1.2–10 its number of pollen foragers or the number of trips per pollen forager and that this effect occurred as soon as the day after treatment (Camazine 1993, Fewell and Bertram 1999, Vaughan and Calderone 2002, Fewell and Page 2000, Janmaat et al. 2000, Weidenmüller and Tautz 2002; see also Rotjan et al. 2002 for the opposite effect with pollen deprivation).

This low evidence of effect was probably due to the very low number of pollen foragers observed in each tunnel on each observation day, with a range between 0 and 11 pollen foragers per 200 flowers. The sampling effort of observing 200 flowers was therefore probably too small given the rarity of the event of observing a bee in a flower during the monitoring. A solution to increase the sample size of pollen foragers could be to observe more flowers per tunnel on each day or to monitor it over more days. Another solution could be to count pollen foragers directly at the hive entrance or to equip the hives with pollen traps. It would also have been relevant to record the amount of pollen carried by nectar foragers on their bodies to confirm the mechanisms of our hypothesis (for methodology, see for example Vaissière and Froissart 1996, Weinman et al. 2023). At last, it would be interesting for the future to repeat this experiment with more days monitored after the pollen supplementation, to know the duration of the effect after treatment on pollen foraging and at which frequency this treatment should be repeated during the flowering of a crop. Dogterom and Winston (1999) showed for instance that colonies supplemented with pollen in the combs recovered levels of pollen foraging similar to colonies not supplemented 7 days after supplementation.

On the other hand, the pollen supplementation in a paste form laid on top of the hive frames did not increase the number of pollen grains on melon stigmas, nor did it decrease the pollen foraging activity of honey bees either. This result can be explained by the fact that, although pollen pastes and pollen substitutes laid on top of the hive frames are usually used and consumed by honey bee colonies, the pollen pastes and substitutes are not stored in the combs (Noordyke et al. 2021). Yet, when they are back to the nest, honey bee pollen foragers assess the colony pollen stores by the time spent and the numbers of cells inspected to find a cell close to unsealed brood with enough available space to unload their pollen pellets (Dreller and Tarpy 2000, Calderone and Johnson 2002). In this way, pollen stores are assessed by direct contact with pollen cells (Dreller and Tarpy 2000, Vaughan and Calderone 2002), and not by scents emitted by pollen (Camazine 1993, Dreller and Tarpy 2000), nor by information exchanged with nonforaging nestmates such as nurses through trophallaxis or antennal contact (Vaughan and Calderone 2002). Consequently, if the cells around the unsealed brood stay empty, the pollen foragers will continue to assess the pollen stores as low, and thus continue to collect pollen.

Likewise, other studies also showed that pollen or protein pastes laid on top of the hive frames did not decrease the pollen foraging activity of colonies (de Mattos et al. 2015, Lamontagne-Drolet et al. 2019, Ahmad et al. 2021), nor increase the pollen stores (Doull 1980a, Kalev et al. 2002, Avni et al. 2009; but see Doull 1973, 1980b). In these studies, the colonies may have been monitored in pollen-deficient environments, like Kalev et al. (2002) which was conducted in tunnels which were pollen-poor environments for bees, i.e., an environment similar to that of our study. In such pollen-poor environments, the pollen stores cannot be increased by a sufficient influx of environmental pollen, this pollen influx being too low to exceed the daily consumption of the pollen stores. However, in pollen-rich environments enabling a colony to have a good supply of environmental pollen, as in the open, we could assume that a colony supplemented with pollen or protein paste on top of its frames might be able to fill a part of its protein needs by consuming the paste, resulting in a decrease in the consumption of its pollen stores, and thus in an increase in its pollen stores. It is probably what happened in Doull (1973, 1980b). The influx of excess pollen into the colony could cause the colony to progressively increase its pollen stores up to the certain homeostatic point at which the colony decreases its pollen foraging activity. As the increase of pollen stores would be gradual, a certain delay period would be expected before the homeostatic point is reached and the colony decreases its pollen foraging activity. It remains to be determined for how long this delay period is necessary before the supplementation of pollen or protein paste on top of the frames decreases the pollen foraging activity of the colony and, if it does, by how much.

Finally, the pollen supplementation in the combs did not increase the fruit set or the fruit mass in our study, except the seed set, while it increased the stigmatic pollen loads. The average seed set we got in our data corresponds to the average number of 500-600 filled seeds found in the literature for well-shaped fruits in open fields or in enclosures (Mann and Robinson 1950, McGregor and Todd 1952, Mann 1953, Iselin et al. 1974, Gaye et al. 1991), and it is above the required minimum of 400 filled seeds to have fruits of marketable size (McGregor and Todd 1952, Bohn and Davis 1964, Fisher and Pomeroy 1989, Vaissière and Froissart 1996). The absence of effect on fruit set and fruit mass was not surprising and could easily be explained by the fact that melon is strongly resource limited, with plants setting into fruits only their first well-pollinated pistillate flowers (Mann and Robinson 1950, Mann 1953, Norden 1985, Valantin et al. 1999, Valantin-Morison et al. 2006, Pitrat 2008). In our study, the pistillate flowers were probably adequately pollinated, even in the control tunnels in which the honey bee colonies were not pollen supplemented. Resource limitation has therefore probably mitigated the positive impact of the increased stigmatic pollen loads resulting from the pollen supplementation in the combs that we observed.

In the end, our study was mainly a proof of concept, and the method of supplementing honey bee colonies with pollen was demonstrated to be relevant for increasing the production and quality of seeds and fruits of melon, and probably of any nectariferous crops displaying anthers isolated from stigmas, whenever such crops present a deficit of seed and fruit production due to pollen limitation. It is also relevant to increase the pollination effectiveness of honey bees, to enable the farmers to decrease their colony stocking rate, thereby saving on input costs, and decreasing (i) the intraspecific competition between colonies for the nectar and pollen resources available in the crop and the environment and (ii) the potential detrimental effects on native flora and entomofauna of importing too many managed colonies at a single location (Geslin et al. 2017b, 2023, Morales et al. 2017, Russo et al. 2021).

To conclude, supplementing honey bee colonies with pollen in the combs can therefore be an effective method for increasing their pollinating activity in nectariferous crops displaying anthers isolated from stigmas. It could be applied to a wide range of crops exhibiting these features, listed in Supplementary Table S1, including for instance avocado, strawberry, Apiaceae, Amaryllidaceae, and Asteraceae seed crops, Cucurbitaceae, and all hybrid seed crops grown with a gynodioecious system of parental lines. This method would be especially effective in pollen-deficient environments such as enclosures. On the other hand, supplementing colonies with pollen paste or pollen substitute on top of the hive frames might be an easier and faster means of increasing their pollinating activity in the open, by providing the protein supplementation early enough before the onset of bloom of the target crop. But this last assumption remains to be tested, and the amount of time needed to get the expected effect after the protein supplementation remains to be assessed. This concept could be extended to other social bee species managed for crop pollination that also decrease their pollen foraging activity following pollen supplementation such as bumble bees (*Bombus impatiens* Cresson [Hymenoptera: Apidae]; Kitaoka and Nieh 2009) and stingless bees (*Melipona subnitida* Ducke [Hymenoptera: Apidae]; Maia-Silva et al. 2016).

## Supplementary Material

toad222_suppl_Supplementary_Tables_S1-S2_Figures_S1-S6

toad222_suppl_Supplementary_Material
