# Supplementary material for: Supplementing honey bee (Hymenoptera: Apidae) colonies with pollen increases their pollinating activity on nectariferous crops with anthers isolated from stigmas
Source: J Econ Entomol. 2023 Dec 13;117(1):43–57. doi: 10.1093/jee/toad222 (PMC10860159; doi:10.1093/jee/toad222)
Supplement: toad222_suppl_Supplementary_Tables_S1-S2_Figures_S1-S6 [file toad222_suppl_supplementary_tables_s1-s2_figures_s1-s6.pdf]

Supplementary material

JOURNAL OF ECONOMIC ENTOMOLOGY

Supplementing honey bee (Hymenoptera: Apidae) colonies with pollen increases their pollinating activity on crops with anthers isolated from stigmas

Stan Chabert, Nicolas Morison, Marie-Josée Buffière, Laurent Guilbaud, Céline Pleindoux, Géraud de Premorel, Philippe Royer, Marie Harruis, Bernard E. Vaissière

Contents

1. Supplementary table and figures
2. Pollen supplementation treatments of honey bee colonies
3. Analysis of stigmatic pollen loads

1. Supplementary table and figures

Table S1. List of the main entomophilous crops displaying a spatial or temporal separation between the sex functions, with both sex functions expressed with nectar secretion. This list was established using McGregor (1976), Klein et al. (2007) and Siopa et al. (2023).

Flower biology	Family	Species name	Common name	Remarks and references
Monoecy	Araceae	*Colocasia esculenta*	Taro	OP seed production; with protogyny within the same inflorescence.
	Arecaceae	*Cocos nucifera*	Coconut	May occasionally have hermaphrodite flowers.
	Cucurbitaceae	*Benincasa hispida*	Wax gourd	
		Citrullus lanatus	Watermelon	Some cultivars are andromonoecious.
		Cucumis melo	Cantaloupe melon, Muskmelon	Some cultivars are andromonoecious.
		Cucumis sativus	Cucumber	Some cultivars are andromonoecious.
		Cucurbita argyrosperma	Cushaw squash	
		Cucurbita maxima	Gourd, Pumpkin, Winter squash	
		Cucurbita moschata	Gourd, Pumpkin, Winter squash	
		Cucurbita pepo	Courgette, Gourd, Pumpkin, Summer squash, Zucchini	
		Luffa acutangula	Ridge gourd	
		Luffa aegyptiaca (syn. *L. cylindrica*)	Sponge gourd	
		Momordica charantia	Bitter gourd, Bitter squash, Bitter melon	
	Euphorbiaceae	*Manihot esculenta*	Cassava	For breeding
Dioecy	Asparagaceae	*Asparagus officinalis*	Asparagus	OP seed production
	Dioscoreaceae	*Dioscorea communis*	Yam	For breeding
	Myrtaceae	*Pimenta dioica*	Allspice	
	Rosaceae	*Rubus chamaemorus*	Cloudberry	Karst et al. (2008); Brown and McNeil (2009); Naess and Chagnon (2011)
	Sapindaceae	*Nephelium lappaceum*	Rambutan	Androdioecious but with functionally female pseudohermaphrodite flowers on bisexual plants, thus considered functionally dioecious (Lan, 1984; Slaa et al., 2006).

Gynodioecy	Amaryllidaceae	*Allium ampeloprasum*	Leek	GMS hybrid seed production (Celebi-Toprak and Alan, 2021; e.g., Fijen et al., 2018, 2020)
		Allium cepa	Onion, shallot	CMS hybrid seed production (Havey, 2018)
		Allium fistulosum	Welsh onion	CMS hybrid seed production
		Allium schoenoprasum	Chives	CMS hybrid seed production
	Apiaceae	*Apium graveolens*	Celery	CMS hybrid seed production
		Daucus carota	Carrot	CMS hybrid seed production (Chen and Liu, 2014; Simon, 2021; e.g., Rodet et al., 1991; Mayer and Lunden, 2001; Gaffney et al., 2011, 2018, 2019)
		Pastinaca sativa	Parsnip	CMS hybrid seed production (Chappell and Dunford, 2021)
	Asteraceae	*Carthamus tinctorius*	Safflower	CMS hybrid seed production (Golkar and Karimi, 2019)
		Cichorium intybus	Chicory	GMS hybrid seed production
		Cynara cardunculus	Cardoon, Globe artichoke	GMS hybrid seed production (Calabrese et al., 2019; e.g., Morison et al., 2000)
		Helianthus annuus	Sunflower	CMS hybrid seed production (Chen and Liu, 2014; Bohra et al., 2016; e.g., Tepedino and Parker, 1982; DeGrandi-Hoffman and Martin, 1993; DeGrandi-Hoffman and Watkins, 2000; Greenleaf and Kremen, 2006; Susic Matin and Farina, 2016; Estravis-Barcala et al., 2019)
	Brassicaceae	*Brassica juncea*	Brown mustard	CMS hybrid seed production (Yamagishi and Bhat, 2014; Singh et al., 2019)
		Brassica napus	Oilseed rape, Canola, Rapeseed	CMS hybrid seed production (Yamagishi and Bhat, 2014; Pelletier and Budar, 2015; Singh et al., 2019; e.g., Waytes et al., 2022; Robinson et al., 2023)
		Brassica oleracea	Cabbage, Cauliflower, Broccoli, Brussel sprouts	CMS hybrid seed production (Yamagishi and Bhat, 2014; Pelletier and Budar, 2015; Singh et al., 2019)
		Brassica rapa (syn. *B. campestris*)	Canola, Turnip, Chinese cabbage	CMS hybrid seed production (Yamagishi and Bhat, 2014; Singh et al., 2019)
		Raphanus sativus	Radish	CMS hybrid seed production (Chen and Liu, 2014; Yamagishi and Bhat, 2014; Singh et al., 2019)
	Cucurbitaceae	*Citrullus lanatus*	Watermelon	GMS hybrid seed production (Robinson, 1999; Dong et al., 2021; Jang et al., 2021)
		Cucumis melo	Cantaloupe melon, Muskmelon	GMS and Gynoecious lines for hybrid seed production (Robinson, 1999; Kumar and Singh, 2004)
		Cucumis sativus	Cucumber	Gynoecious lines for hybrid seed production (Robinson, 1999; Kumar and Singh, 2004)
		Cucurbita maxima	Gourd, Pumpkin, Winter squash	GMS lines and application of PGH for hybrid seed production (Robinson, 1999; Kumar and Singh, 2004)
		Cucurbita moschata	Gourd, Pumpkin, Winter squash	Application of PGH for hybrid seed production (Robinson, 1999; Kumar and Singh, 2004)
		Cucurbita pepo	Courgette, Gourd, Pumpkin, Summer squash, Zucchini	Application of PGH for hybrid seed production (Robinson, 1999; Kumar and Singh, 2004)

	Luffa acutangula	Ridge gourd	CGMS hybrid seed production (Pradeepkumar et al., 2018)
	Momordica charantia	Bitter gourd, Bitter squash, Bitter melon	Gynoecious lines for hybrid seed production (Behera et al., 2009)
Fabaceae	*Cajanus cajan*	Pigeon pea	CMS hybrid seed production (Bohra et al., 2016)
	Glycine max	Soybean	CMS hybrid seed production (Palmer et al., 2010; Bohra et al., 2016)
	Medicago sativa	Alfalfa	CMS hybrid seed production
	Phaseolus vulgaris	Common bean	CMS hybrid seed production (Chen and Liu, 2014; Bohra et al., 2016)
	Vicia faba	Faba bean	CMS hybrid seed production (Bohra et al., 2016)
Malvaceae	*Gossypium hirsutum*	Upland cotton	CMS hybrid seed production (Bohra et al., 2016; e.g., Vaissière, 1991)
Solanaceae	*Capsicum annuum*	Pepper	CMS hybrid seed production (Chen and Liu, 2014)

Dichogamy: Protogyny	Apiaceae	*Arracacia Xanthorrhiza*	Arracacha	OP seed production (protogyny reported in Rander Knudsen et al., 2006)
	Sapotaceae	*Vitellaria paradoxa*	Shea	Lassen et al. (2018a); Stout et al. (2018); Delaney et al. (2020)
Dichogamy: Protandry	Amaryllidaceae	*Allium ampeloprasum*	Leek	OP seed production
		Allium cepa	Onion, shallot	OP seed production
		Allium fistulosum	Welsh onion	OP seed production
		Allium sativum	Garlic	For breeding
		Allium schoenoprasum	Chives	OP seed production
	Apiaceae	*Anthriscus cerefolium*	Chervil	OP seed production
		Apium graveolens	Celery	OP seed production
		Carum carvi	Caraway	
		Coriandrum sativum	Coriander	
		Daucus carota	Carrot	OP seed production
		Foeniculum vulgare	Fennel	OP seed production
		Pastinaca sativa	Parsnip	OP seed production
		Petroselinum crispum	Parsley	OP seed production
	Asparagaceae	*Agave fourcroydes*	Henequen	OP seed production
		Agave sisalana	Sisal	OP seed production
	Asteraceae	*Carthamus tinctorius*	Safflower	
		Cichorium intybus	Chicory	OP seed production
		Cynara cardunculus	Cardoon, Globe artichoke	OP seed production
		Guizotia abyssinica	Niger, Noug	
		Helianthus annuus	Sunflower	But requires only a low bee density to be fully pollinated (Chabert et al., 2022), so probably no need to supplement colonies with pollen.
		Helianthus tuberosus	Jerusalem artichoke, Topinambur	OP seed production
	Fabaceae	*Parkia biglobosa*	West African parkland tree, African locust bean	Lassen et al. (2018b)
		Scorzonera hispanica	Black salsify	OP seed production
	Proteaceae	*Macadamia* spp.	Macadamia	Anthesis progresses basipetally or acropetally along racemes, from the center or both ends, depending on the cultivar and environment conditions (Trueman, 2013). The flowers at the staminate stage are therefore spatially separated from the flowers at the pistillate stage on the racemes, and bee pollen foragers may collect pollen on the staminate flowers without contacting the pistillate flowers.

Heterodichogamy	Lauraceae	*Persea americana*	Avocado	Displays protogynous heterodichogamy: the individuals of one morph (cultivar A) bear hermaphrodite flowers beginning their anthesis in the morning with the pistillate phase and pursuing it in the afternoon with the staminate phase, whereas the individuals of the other morph (cultivar B) bear hermaphrodite flowers beginning their anthesis in the afternoon with the pistillate phase and pursuing it the next morning with the staminate phase (Renner, 2001; Endress, 2010). Honey bee pollen foragers have been reported to be pollen thieves (Ish-Am and Eisikowitch, 1993)
	Rhamnaceae	*Ziziphus jujuba*	Jujube	Displays protandrous heterodichogamy: the individuals of one morph (cultivar A) bear hermaphrodite flowers beginning their anthesis in the morning with the staminate phase and pursuing it in the afternoon with the pistillate phase, whereas the individuals of the other morph (cultivar B) bear hermaphrodite flowers beginning their anthesis in the afternoon with the staminate phase and pursuing it the next morning with the pistillate phase (Lyrene, 1983; Yao et al., 2015; Wang et al., 2021; Tel-Zur, 2023).
Duodichogamy	Sapindaceae	*Dimocarpus longan*	Longan	Flowering sequence in the inflorescences: first, anthesis of functionally male pseudohermaphrodite flowers, then functionally female pseudohermaphrodite flowers, and finally functionally male pseudohermaphrodite flowers (Lora et al., 2018).
		Litchi chinensis	Lychee	Flowering sequence in the inflorescences: first, anthesis of functionally male pseudohermaphrodite flowers, then functionally female pseudohermaphrodite flowers, and finally functionally male pseudohermaphrodite flowers (Menzel, 1984).
Herkogamy	Rosaceae	*Fragaria × ananassa*	Strawberry	Stamens are spatially distributed around the gynoecium in an independent whorl. If bee pollen foragers can collect pollen without contacting the stigmas, they could hamper the pollinating activity of nectar foragers. But this remains an assumption for this crop; it needs to be verified that pollen foragers can collect pollen without contacting the stigmas.

Definitions

(Pseudo)hermaphrodite: adjective used to describe a flower that is bisexual, i.e., that bears both the two sex functions, the pistil, responsible for the female function, and the stamens, responsible for the male function, or to describe an individual that bears bisexual flowers. A pseudohermaphrodite flower has one of its sex functions that is dysfunctional.

Pistillate flower: unisexual flower that bears only a functional pistil. These flowers are functionally female.

Staminate flower: unisexual flower that bears only functional stamens. These flowers are functionally male.

Monoecy: monoecious individuals bear separate pistillate flowers and staminate flowers.

Andromonoecy: andromonoecious individuals bear staminate flowers and hermaphrodite flowers.

Dioecy: dioecious species are composed of female individuals that bear only pistillate flowers and of male individuals that bear only staminate flowers.

Gynodioecy: gynodioecious species are composed of female individuals that bear only pistillate flowers and of bisexual individuals that bear hermaphrodite flowers or pistillate flowers and staminate flowers.

Dichogamy: dichogamous flowers are uni- or bisexual flowers with temporal separation between sex phases within a flower or within an individual.

Protogyny: protogynous flowers are hermaphrodite flowers with a pistillate phase preceding a staminate phase.

Protandry: protandrous flowers are hermaphrodite flowers with a staminate phase preceding a pistillate phase.

Heterodichogamy: synchronized dichogamy in which there are two morphs of individuals with reciprocal flowering behaviors (Endress, 2010).

Duodichogamy: synchronized flowering sequence on an individual, with two distinct staminate phases separated by a pistillate phase, i.e., a male-female-male flowering sequence (Pauly et al., 2023).

Herkogamy: hermaphrodite flowers in which the stigma is spatially separated from the anthers.

OP seed production: open pollinated seed production. The benefit of insect pollination is involved only in the seed production used to grow the plants on which the vegetative parts will be harvested for human or animal consumption. It is the cultivation of the plant generation preceding the cultivation of the plants used for human or animal consumption. 'Open pollinated' means that the seeds are produced from the same plant population as that used for human or animal consumption. These cultivars are called 'open pollinated cultivars' or 'population cultivars'.

Hybrid seed production: like with OP seed production, these crops are used to produce the seeds to grow the plant generation on which the vegetative parts or the seeds will be harvested for human or animal consumption. They are also the cultivation of the plant generation preceding the cultivation of the plants used for human or animal consumption. 'Hybrid' means that the seeds are produced from the cross between two inbred lines, a male-sterile line (bearing pistillate flowers with anthers devoid of pollen) on which the F_1 hybrid seed is harvested, and a male-fertile line (bearing hermaphrodite flowers) which is used to provide the pollen for pollination. This crop system is equivalent to a gynodioecious system. The male-sterile lines are obtained either with cytoplasmic male sterility (**CMS**), genic male sterility (**GMS**), cytoplasmic-genic male sterility (**CGMS**), or plant growth hormones (**PGH**) (Kumar and Singh, 2004; Chase, 2007; Chase et al., 2010; Kempe and Gils, 2011; Chen and Liu, 2014). The cultivars obtained from the crosses between the two inbred lines are called 'hybrid cultivars' or 'F_1 hybrid cultivars'. The crop species listed for the hybrid seed

productions are mainly based on Havey (2004) and Colombo and Galmarini (2017). Some other references are quoted to describe the CMS or GMS systems of some crop species, and some examples of pollination studies are quoted for some crop species.

<u>For breeding</u>: the benefit of insect pollination is involved in the seed production used for plant breeding, and the cultivars are reproduced vegetatively.

References

Behera TK, Dey SS, Munshi AD, Gaikwad AB, Pal A, Singh I. 2009. Sex inheritance and development of gynoecious hybrids in bitter gourd (*Momordica charantia* L.). *Scientia Horticulturae*, *120*(1), 130-133.

Bohra A, Jha UC, Adhimoolam P, Bisht D, Singh NP. 2016. Cytoplasmic male sterility (CMS) in hybrid breeding in field crops. *Plant Cell Reports*, 35, 967-993.

Brown AO, McNeil JN. 2009. Pollination ecology of the high latitude, dioecious cloudberry (*Rubus chamaemorus*; Rosaceae). *American Journal of Botany*, 96(6), 1096-1107.

Calabrese N, Cravero V, Pagnotta MA. 2019. *Cynara cardunculus* propagation. In: Portis E, Acquadro A, Lanteri S. (eds.), *The Globe Artichoke Genome*. Springer, pp. 21-40.

Celebi-Toprak F, Alan AR. 2021. Genetic improvement of leek (*Allium ampeloprasum* L.). In: Al-Khayri JM, Jain SM, Johnson DV. (eds.), *Advances in Plant Breeding Strategies: Vegetable Crops. Volume 8: Bulbs, Roots and Tubers*. Springer, pp. 51-97.

Chabert S, Mallinger RE, Senechal C, Fougeroux A, Geist O, Guillemard V, Leylavergne S, Malard C, Pousse J, Vaissiere BE. 2022. Importance of maternal resources in pollen limitation studies with pollinator gradients: A case study with sunflower. *Agriculture, Ecosystems & Environment*, 330, 107887.

Chappell LH, Dunford AJ. 2021. Parsnip (*Pastinaca sativa* L.) breeding for the future. In: Al-Khayri JM, Jain SM, Johnson DV. (eds.), *Advances in Plant Breeding Strategies: Vegetable Crops. Volume 8: Bulbs, Roots and Tubers*. Springer, pp. 239-273.

Chase CD. 2007. Cytoplasmic male sterility: a window to the world of plant mitochondrial-nuclear interactions. *Trends in Genetics*, 23(2), 81-90.

Chase CD, Ribarits A, Heberle-Bors E. 2010. Male sterility. In: Pua EC, Davey MR. (eds.), *Plant Developmental Biology - Biotechnological Perspectives. Volume 1*. Springer, pp. 437-457.

Chen L, Liu YG. 2014. Male sterility and fertility restoration in crops. *Annual Review of Plant Biology*, 65, 579-606.

Colombo N, Galmarini CR. 2017. The use of genetic, manual and chemical methods to control pollination in vegetable hybrid seed production: a review. *Plant Breeding*, 136(3), 287-299.

DeGrandi-Hoffman G, Martin JH. 1993. The size and distribution of the honey bee (*Apis mellifera* L.) cross-pollinating population on male-sterile sunflowers (*Helianthus annuus* L.). *Journal of Apicultural Research*, 32(3-4), 135-142.

DeGrandi-Hoffman G, Watkins JC. 2000. The foraging activity of honey bees *Apis mellifera* and non-*Apis* bees on hybrid sunflowers (*Helianthus annuus*) and its influence on cross-pollination and seed set. *Journal of Apicultural Research*, 39(1-2), 37-45.

Delaney A, Dembele A, Nombré I, Gnane Lirasse F, Marshall E, Nana A, Vickery J, Tayleur C, Stout JC. 2020. Local-scale tree and shrub diversity improves pollination services to shea trees in tropical West African parklands. *Journal of Applied Ecology*, 57(8), 1504-1513.

Dong W, Wu D, Yan C, Wu D. 2021. Mapping and analysis of a novel genic male sterility gene in watermelon (*Citrullus lanatus*). *Frontiers in Plant Science*, 12, 639431.

Endress PK. 2010. The evolution of floral biology in basal angiosperms. *Philosophical Transactions of the Royal Society B: Biological Sciences*, 365(1539), 411-421.

Estravis Barcala MC, Palottini F, Farina WM. 2019. Honey bee and native solitary bee foraging behavior in a crop with dimorphic parental lines. *PloS one*, 14(10), e0223865.

Fijen TP, Scheper JA, Boom TM, Janssen N, Raemakers I, Kleijn D. 2018. Insect pollination is at least as important for marketable crop yield as plant quality in a seed crop. *Ecology Letters*, 21(11), 1704-1713.

Fijen TP, Scheper JA, Vogel C, van Ruijven J, Kleijn D. 2020. Insect pollination is the weakest link in the production of a hybrid seed crop. *Agriculture, Ecosystems & Environment*, 290, 106743.

Gaffney A, Allen GR, Brown PH. 2011. Insect visitation to flowering hybrid carrot seed crops. *New Zealand Journal of Crop and Horticultural Science*, 39(2), 79-93.

Gaffney A, Bohman B, Quarrell SR, Brown PH, Allen GR. 2018. Frequent insect visitors are not always pollen carriers in hybrid carrot pollination. *Insects*, 9(2), 61.

Gaffney A, Bohman B, Quarrell SR, Brown PH, Allen GR. 2019. Limited cross plant movement and non-crop preferences reduce the efficiency of honey bees as pollinators of hybrid carrot seed crops. *Insects*, 10(2), 34.

Golkar P, Karimi S. 2019. Safflower (*Carthamus tinctorius* L.) breeding. In: Al-Khayri JM, Jain SM, Johnson DV. (eds.), *Advances in Plant Breeding Strategies: Industrial and Food Crops. Volume 6*. Springer, pp. 537-575.

Greenleaf SS, Kremen C. 2006. Wild bees enhance honey bees' pollination of hybrid sunflower. *Proceedings of the National Academy of Sciences*, 103(37), 13890-13895.

Havey MJ. 2004. The use of cytoplasmic male sterility for hybrid seed production. In: Daniell H, Chase C. (eds.), *Molecular Biology and Biotechnology of Plant Organelles: Chloroplasts and Mitochondria*. Springer, pp. 623-634.

Havey MJ. 2018. Onion breeding. *Plant Breeding Reviews*, 42, 39-85.

Ish-Am G, Eisikowitch D. 1993. The behaviour of honey bees (*Apis mellifera*) visiting avocado (*Persea americana*) flowers and their contribution to its pollination. *Journal of Apicultural Research*, 32(3-4), 175-186.

Jang YJ, Sim TY, Ryu J, Rhee SJ, Kim Y, Lee GP. 2021. Identification of a candidate locus and development of a molecular marker for male sterility in watermelon. *Horticultural Science and Technology*, *39*(5), 673-683.

Karst AL, Antos JA, Allen GA. 2008. Sex ratio, flowering and fruit set in dioecious *Rubus chamaemorus* (Rosaceae) in Labrador. *Botany*, 86(2), 204-212.

Kempe K, Gils M. 2011. Pollination control technologies for hybrid breeding. *Molecular Breeding*, 27, 417-437.

Klein AM, Vaissière BE, Cane JH, Steffan-Dewenter I, Cunningham SA, Kremen C, Tscharntke T. 2007. Importance of pollinators in changing landscapes for world crops. *Proceedings of the Royal Society B: Biological Sciences*, 274(1608), 303-313.

Kumar S, Singh PK. 2004. Mechanisms for hybrid development in vegetables. *Journal of New Seeds*, *6*(4), 381-407.

Lan LA. 1984. The reproductive biology of rambutan, *Nephelium lappaceum* L. (Sapindaceae). *Gardens Bulletin*, 37, 181-192.

Lassen KM, Kjær ED, Ouédraogo M, Dupont YL, Nielsen LR. 2018b. Controlled pollinations reveal self-incompatibility and inbreeding depression in the nutritionally important parkland tree, *Parkia biglobosa*, in Burkina Faso. *Journal of Pollination Ecology*, 24, 144-156.

Lassen KM, Nielsen LR, Lompo D, Dupont YL, Kjær ED. 2018a. Honey bees are essential for pollination of *Vitellaria paradoxa* subsp. *paradoxa* (Sapotaceae) in Burkina Faso. *Agroforestry Systems*, 92, 23-34.

Lora J, Pham VT, Hormaza JI. 2018. Genetics and breeding of fruit crops in the Sapindaceae family: Lychee (*Litchi chinensis* Sonn.) and longan (*Dimocarpus longan* Lour.). In: Al-Khayri JM, Jain SM, Johnson DV. (eds.), *Advances in Plant Breeding Strategies: Fruits, Volume 3*. Springer, pp. 953-973.

Lyrene PM. 1983. Flowering and fruiting of Chinese jujubes in Florida. *HortScience*, 18(2), 208-209.

Mayer DF, Lunden JD. 2001. Honey bee management and wild bees for pollination of hybrid onion seed. *Acta Horticulturae*, 561, 275-278.

McGregor SE. 1976. *Insect Pollination of Cultivated Crop Plants*. Agriculture Handbook No. 496. Agricultural Research Service, United States Department of Agriculture.

Menzel CM. 1984. The pattern and control of reproductive development in lychee: a review. *Scientia Horticulturae*, 22(4), 333-345.

Morison N, Vaissiere BE, Martin F, Pecaut P, Cambon G. 2000. Pollination of the globe artichoke (*Cynara scolymus* L.) by honey bees (*Apis mellifera* L.) to produce hybrid seed under enclosure. *Apidologie*, 31(1), 115-128.

Naess SK, Chagnon M. 2011. Honeybees are useful as pollinators of the dioecious cloudberry, a high-value northern berry. *Acta Agriculturae Scandinavica Section B - Soil & Plant Science*, 61(1), 1-7.

Palmer RG, Gai J, Sun H, Burton JW. 2010. Production and evaluation of hybrid soybean. *Plant Breeding Reviews*, 21, 263-299.

Pauly G, Larue C, Petit RJ. 2023. Adaptive function of duodichogamy: Why do chestnut trees have two pollen emission peaks?. *American Journal of Botany*, 110(8), e16204.

Pelletier G, Budar F. 2015. *Brassica* Ogu-INRA cytoplasmic male sterility: an example of successful plant somatic fusion for hybrid seed production. In: Li XQ, Donnelly DJ, Jensen TG. (eds.), *Somatic Genome Manipulation: Advances, Methods and Applications*. Springer, New York, pp. 199–216.

Pradeepkumar T, Minimol JS, Mathew D, Veni K, Roch CV, Chithira PG, Unni R. 2018. Development of CGMS system in ridge gourd [*Luffa acutangula* (Roxb.) L.] for production of F_1 hybrids. *Euphytica*, 214, 1-9.

Randers Knudsen S, Ørting B, Sørensen M. 2006. Multiplicación y conservación de arracacha (*Arracacia xanthorrhiza* Bancr.) y ajipa (*Pachyrhizus ahipa* (Wedd.) Parodi). *Botánica económica de los Andes Centrales*, 483-508.

Renner SS. 2001. How common is heterodichogamy?. *Trends in Ecology & Evolution*, 16(11), 595-597.

Robinson RW. 1999. Rationale and methods for producing hybrid cucurbit seed. *Journal of New Seeds*, *1*(3-4), 1-47.

Robinson SV, Cartar RV, Pernal SF, Waytes R, Hoover SE. 2023. Bee visitation, pollination service, and crop yield in commodity and hybrid seed canola. *Agriculture, Ecosystems & Environment*, 347, 108396.

Rodet G, Torre Grossa JP, Bonnet A. 1991. Foraging behavior of *Apis mellifera* L. on male-sterile and male-fertile inbred lines of carrot (*Daucus carota* L.) in gridded enclosures. *Acta Horticulturae*, 288, 371-375.

Simon PW. 2021. Carrot (*Daucus carota* L.) breeding. In: Al-Khayri JM, Jain SM, Johnson DV. (eds.), *Advances in Plant Breeding Strategies: Vegetable Crops. Volume 8: Bulbs, Roots and Tubers*. Springer, pp. 213-238.

Singh S, Dey SS, Bhatia R, Kumar R, Behera TK. 2019. Current understanding of male sterility systems in vegetable *Brassica*s and their exploitation in hybrid breeding. *Plant Reproduction*, 32, 231-256.

Siopa C, Carvalheiro L, Castro H, Loureiro J, Castro S. 2023. Quantifying crop pollinator dependence values - an updated compilation and discussion on methodological approaches. *Authorea*, preprint.

Slaa EJ, Chaves LAS, Malagodi-Braga KS, Hofstede FE. 2006. Stingless bees in applied pollination: practice and perspectives. *Apidologie*, 37(2), 293-315.

Stout JC, Nombre I, de Bruijn B, Delaney A, Doke DA, Gyimah T, et al. 2018. Insect pollination improves yield of Shea (*Vitellaria paradoxa* subsp. *paradoxa*) in the agroforestry parklands of West Africa. *Journal of Pollination Ecology*, 22, 11-20.

Susic Martin C, Farina WM. 2016. Honeybee floral constancy and pollination efficiency in sunflower (*Helianthus annuus*) crops for hybrid seed production. *Apidologie*, 47, 161-170.

Tel Zur N. 2023. Preventing self-fertilization: Insights from *Ziziphus* species. *Frontiers in Plant Science*, 14, 1226502.

Tepedino VJ, Parker FD. 1982. Interspecific differences in the relative importance of pollen and nectar to bee species foraging on sunflowers. *Environmental Entomology*, 11(1), 246-250.

Trueman SJ. 2013. The reproductive biology of macadamia. *Scientia Horticulturae*, 150, 354-359.

Vaissière BE. 1991. Honey bees, *Apis mellifera* L. (Hymenoptera: Apidae), as pollinators of upland cotton, *Gossypium hirsutum* L. (Malvaceae), for hybrid seed production. PhD thesis, Texas A&M University.

Wang F, Sun X, Dong J, Cui R, Liu X, Li X, Wang H, He T, Zheng P, Wang R. 2021. A primary study of breeding system of *Ziziphus jujuba* var. *spinosa*. *Scientific Reports*, 11(1), 10318.

Waytes R, Cartar R, Hoover S. 2022. Consequences of pollinator availability and effectiveness for pollen transfer in a gynodioecious seed crop system. *Journal of Pollination Ecology*, 32, 154-169.

Yamagishi H, Bhat SR. 2014. Cytoplasmic male sterility in Brassicaceae crops. *Breeding Science*, 64(1), 38-47.

Yao S, Huang J, Heyduck R. 2015. Jujube (*Ziziphus jujuba* Mill.) flowering and fruiting in the Southwestern United States. *HortScience*, 50(6), 839-846.

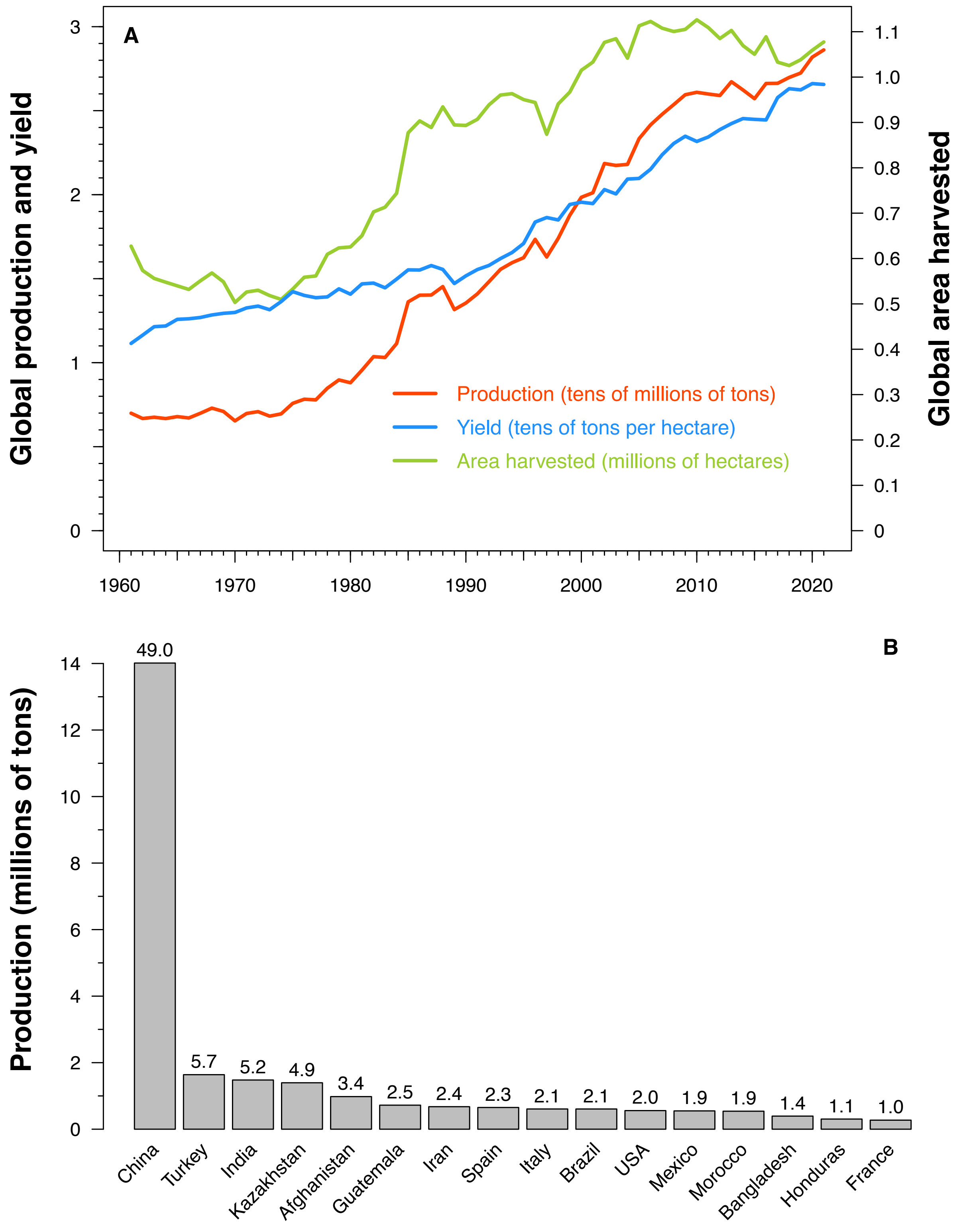

Supplementary Figure S1. (A) Evolution of the global production, yield and area harvested of cantaloupe melon since 1961. (B) Production of cantaloupe melon of the 16 countries yielding more than 1% of the global production in 2021. Numbers above bars are the % of the global production for each country. Data from FAOSTAT (2023).

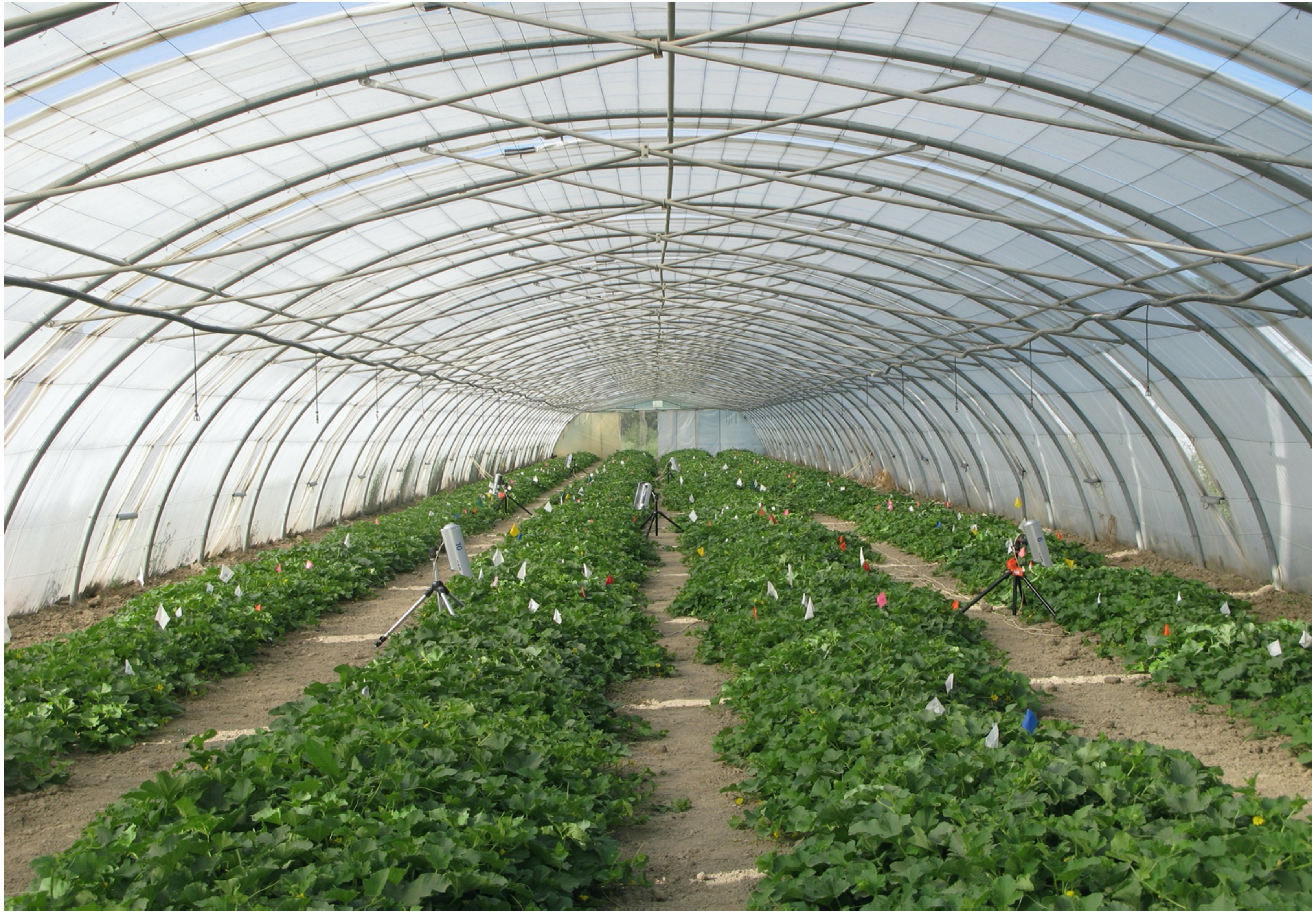

Supplementary Figure S2. Experimental tunnel with the four rows of European cantaloupe melon cv. ‘Neo’. The small colored flags located the pistillate flowers randomly chosen and evenly distributed in the tunnel for which the stigma and fruit were collected. The cameras were used for another experiment. Photo credit: Nicolas Morison/INRAE.

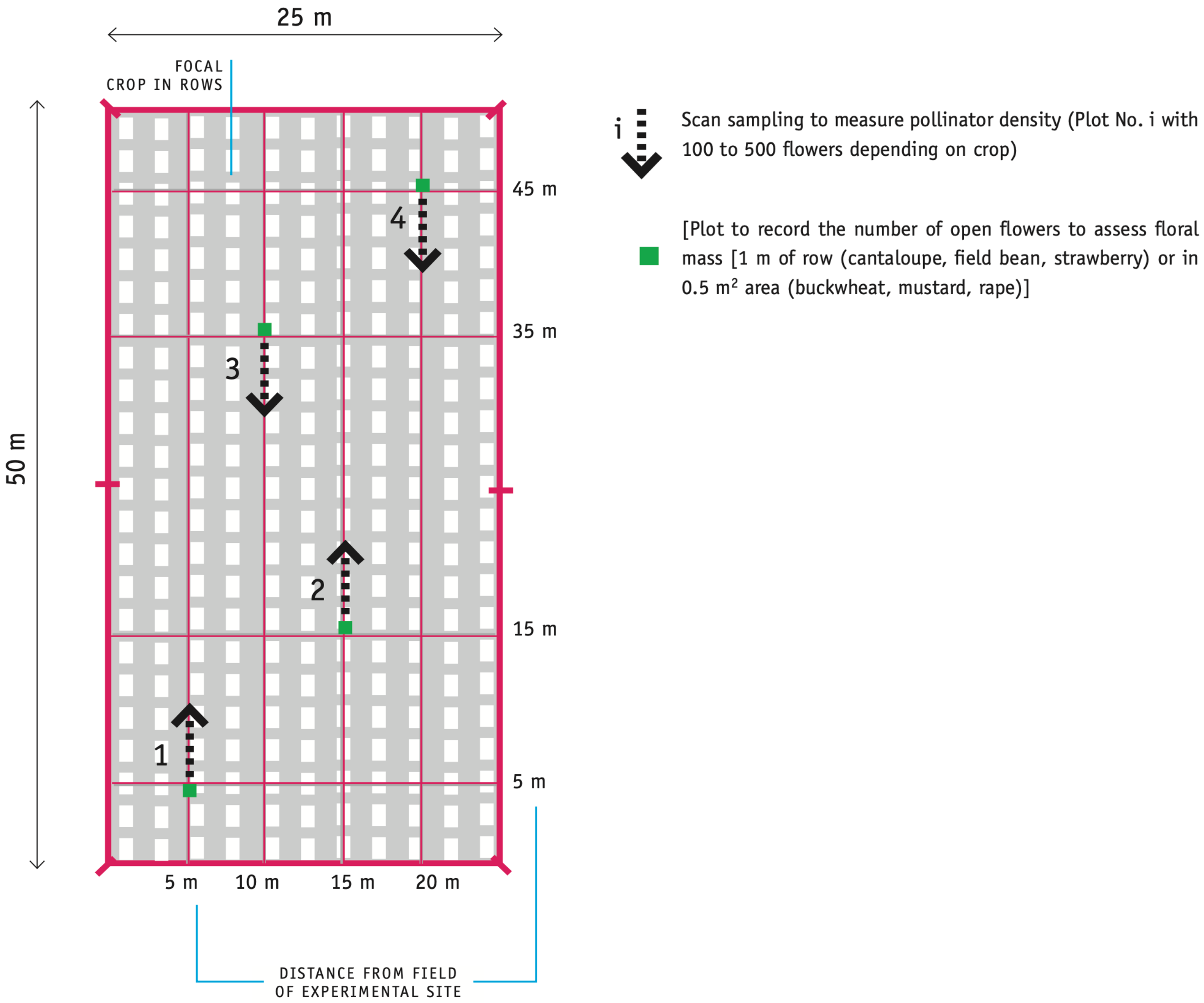

Supplementary Figure S3. Experimental design of the four plots of flower count and transects of honey bee count. Reproduction of Figure 6.1 from Vaissière et al. 2011. Reproduced with permission from © FAO 2011.

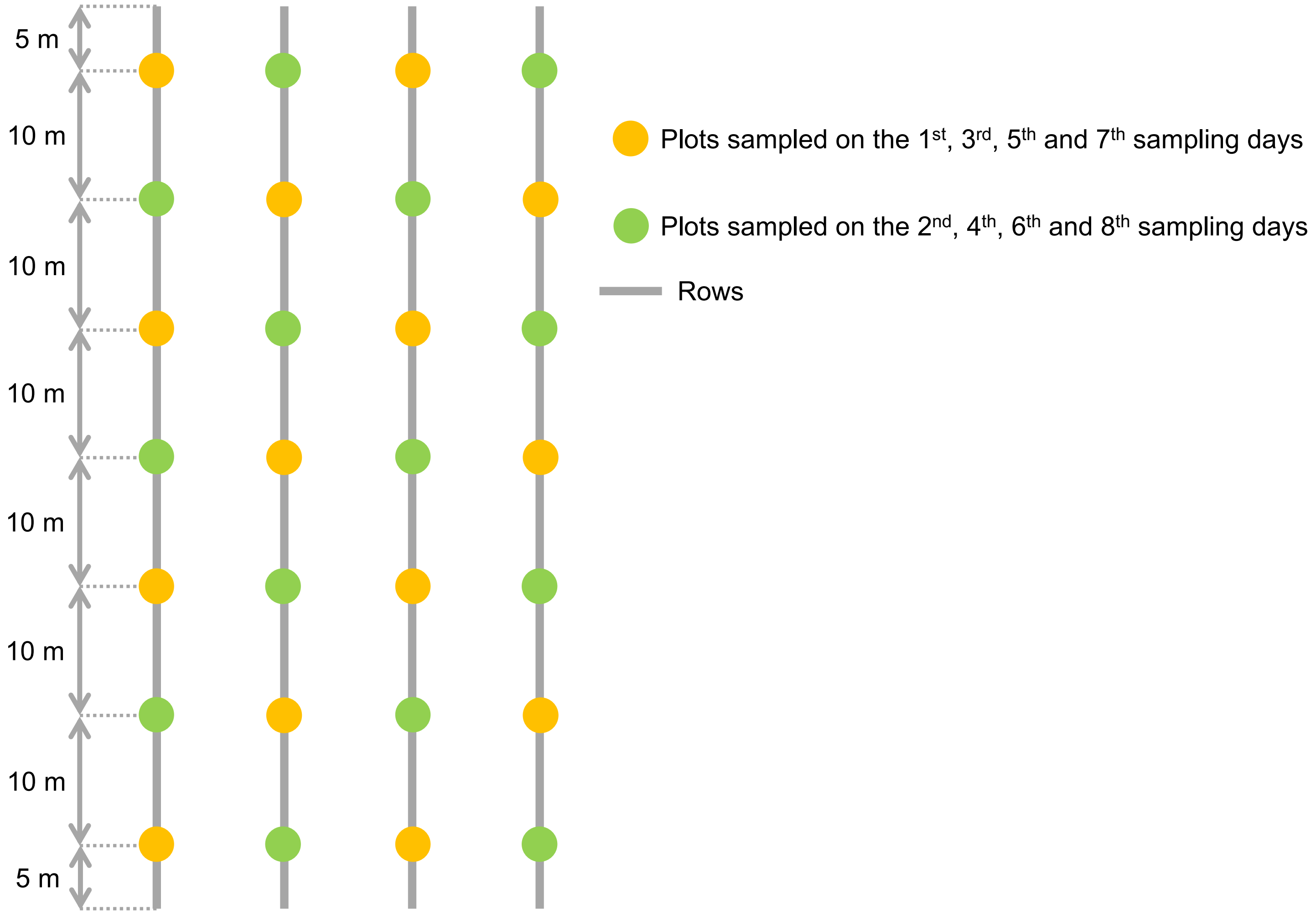

Supplementary Figure S4. Experimental design of the 28 plots sampled every two sampling days to select the pistillate flowers from which the stigmas and fruits were harvested.

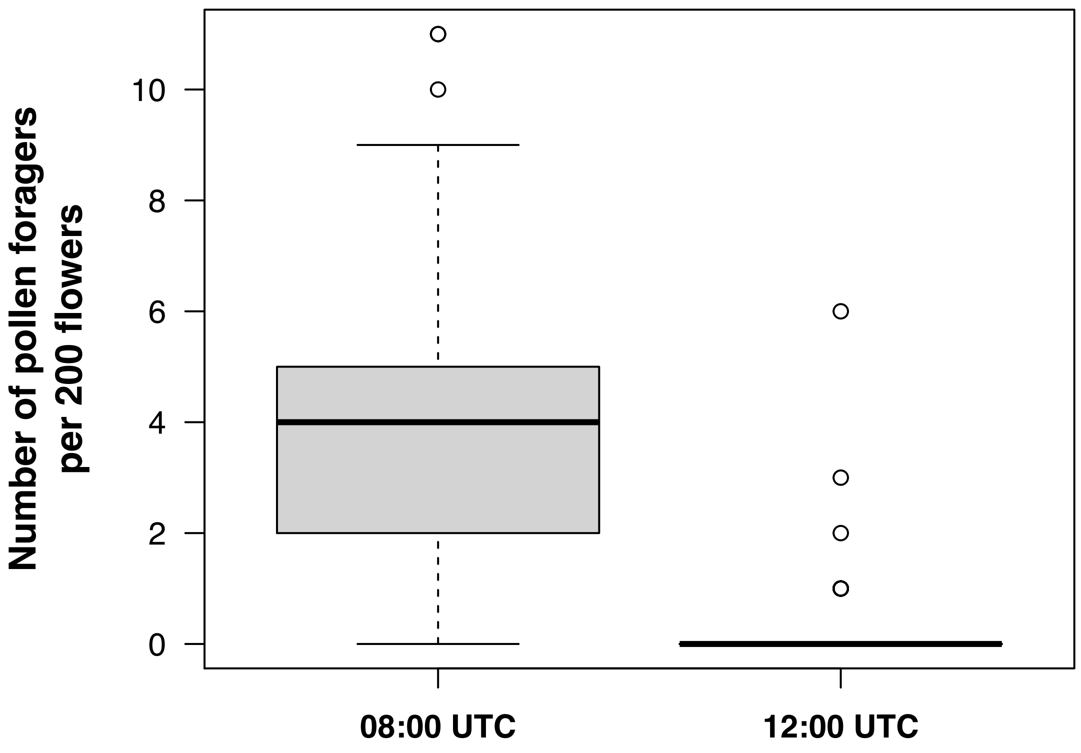

Supplementary Figure S5. Number of pollen foragers counted on 200 flowers around 08:00 UTC (10 AM) and 12:00 UTC (2 PM) in the six experimental tunnels. Each box plot represents, from bottom to top, the minimum, the first quartile, the median (thick line), the third quartile and the maximum. Circles are outliers.

2. Pollen supplementation treatments of honey bee colonies

Commercial fresh pollen pellets, without specified composition except that they did not include melon pollen so as not to interfere with the experiment, were purchased frozen from a beekeeper, and inserted and packed down in combs by hand, before spraying 50% concentrated saccharose syrup (w/v%) on combs to prevent pollen from falling when making the frames upright in the hive (Figure 1). One empty frame side could hold *ca.* 500 g of pollen when filled (1,134 cm^2), so two frames filled on one side were added in the hive on either side of the frames containing brood, the side containing pollen directly facing the brood.

The pollen paste was made of 1 kg of bee pollen pellets mixed with 300 mL of 50% concentrated saccharose syrup (w/v%) made with icing sugar heated to 40°C. The paste was put to cool in a fridge and then spread into a layer of 46 x 20 x 1 cm to cover the whole area on top of the hive frames (Figure 2). The layer of paste was covered on top with baking paper to prevent it from drying out.

Since a honey bee cell measures 5.3 mm in diameter in marketed wax combs (Saucy, 2014), i.e. the cell density is *ca.* 4.54 per cm^2 of combs, that a larva of a honey bee worker ingests *ca.* 150 mg of pollen over its six days of development (Hrassnigg and Crailsheim, 2005), equivalent to the ingestion of *ca.* 25 mg of pollen a day for two thirds of the unsealed brood (made up of *ca.* one third of eggs and two thirds of larvae), and that an adult honey bee worker ingests an average of 3.7 mg of pollen per day (Crailsheim et al., 1992), the average daily pollen consumption was assessed for each colony (Supplementary Table S2). The colony consuming the most pollen is supposed to ingest *ca.* 43 g of pollen a day, so that the 1 kg of pollen added to the colony is equivalent to more than three weeks of pollen consumption. Yet a honey bee colony maintains a sufficient pollen reserve to meet its needs for about a ten days (Seeley, 1995). Thus, we expected that the amount of 1 kg of pollen added to the colonies was large enough to exceed the potential homeostatic set point beyond which the colonies decrease their pollen collection.

References

Crailsheim K, Schneider LHW, Hrassnigg N, Bühlmann G, Brosch U, Gmeinbauer R, and Schöffmann B. 1992. Pollen consumption and utilization in worker honeybees (*Apis mellifera carnica*): dependence on individual age and function. *Journal of Insect Physiology*, *38*(6), 409-419.

Hrassnigg N, and Crailsheim K. 2005. Differences in drone and worker physiology in honeybees (*Apis mellifera*). *Apidologie*, *36*(2), 255-277.

Saucy F. 2014. On the natural cell size of European honey bees: a "fatal error" or distortion of historical data?. *Journal of Apicultural Research*, *53*(3), 327-336.

Seeley TD. 1995. The foraging abilities of a colony. In: Seeley TD (ed.), *The Wisdom of the Hive: the Social Physiology of Honey Bee Colonies*. Harvard University Press, pp. 46-68.

Supplementary Table S2. Features of the honey bee colonies introduced in the experimental tunnels of each repetition.

Repetition	1st repetition			2nd repetition		
Colony treatment	Control	Pollen paste	Pollen in the combs	Control	Pollen paste	Pollen in the combs
Population size[a]	3,700	7,300	8,200	8,200	9,700	11,300
Unsealed brood area (cm^2)	117	70	64	0	68	18
Sealed brood area (cm^2)	126	139	136	196	86	42
Honey store area (cm^2)	157	138	296	133	143	189
Pollen store area (cm^2)	47	0	17	0	0	0
Estimated daily pollen consumption of adult workers (g)	13.7	27.0	30.3	30.3	35.9	41.8
Estimated daily pollen consumption of unsealed brood (g)	8.8	5.3	4.8	0	5.1	1.4
Estimated total daily pollen consumption of colonies (g)	22.5	32.3	35.2	30.3	41.0	43.2

[a]Population size: number of adult worker honey bees

3. Analysis of stigmatic pollen loads

Extraction, staining, filtration and scanning of the pollen loads from dry stigmas

The methodology which consists of removing pollen grains from dry stigmas by sonication was developed by Vaissière (1991) on upland cotton *Gossypium hirsutum*. It enables one to analyse the stigmatic pollen loads easily and with a good reliability as all the pollen grains are removed from the stigmas without breaking them. This methodology was used by Vaissière and Froissart (1996) on *Cucumis melo*, and also reported in Dafni (1992) and in Dafni *et al.* (2005).

The dry stigmas of *C. melo* were stored in Eppendorf® tubes until analysis. For stigma analysis, 1.5 mL of an aqueous solution of 1% NaCl (w/v%) and 3 µL of an aqueous solution of 0.1% malachite green (w/v%) were added in each Eppendorf® tube. The two aqueous solutions were both previously filtered on a piece of nylon membrane of 1 µm mesh size (Nitex 03-1/1; Sefar, Heiden, Switzerland) using a standard all-glass 47 mm filtration system (Millipore XX1014700; MilliporeSigma, Darmstadt, Germany). The stigmas were soaked in the Eppendorf® tubes for *ca*. 20 h at ambient temperature using a tube rotator. The content of each tube, including the stigma and the aqueous solution of NaCl and malachite green, was then transferred individually to a 16 mL glass shell vial (60965-4; Kimble®, Rockwood, TN, USA), before adding 10 mL of the 1% NaCl (w/v%) solution and 20 µL of the 0.1% malachite green (w/v%) into the vial. Each vial was then treated a first time with its content with an ultrasonic probe of Ø12.7 mm (Q700 Sonicator; QSonica®, Newtown, CT, USA) vibrating at 20 kHz with an amplitude of 48 µm (40%) during 90 s, the tip of the probe being located 5 mm above the bottom of the vial at rest, to extract pollen grains from the stigma. Stigmas were then examined crushed between two glass slides under a stereo microscope at 50x magnification to make sure that all pollen grains were removed. The pollen suspension in the vial was then treated a second time with the same ultrasonic probe and the same amplitude during 90 s to break any remaining pieces of stigmatic papillae. Pollen grains in the resulting suspension were recovered by successive filtrations on two or three pieces of nylon membrane of 30 µm mesh size (Nitex 03-30/18; Sefar, Heiden, Switzerland), using an all-glass 25 mm filtration system (Millipore XX1012530; MilliporeSigma, Darmstadt, Germany). The ultrasonic probe, the glass shell vials, the glass funnel and the Eppendorf® tube were all rinsed above the filtration system with a small jet of the 1% NaCl solution to recover the potential remaining pollen grains, before cleaning them during 3 min in an ultrasonic cleaning bath (Bransonic® Ultrasonic M Cleaning Bath 5800; Branson®, Danbury, CT, USA). These cleanings avoided any pollen contamination between successive samples. The pieces of nylon membrane of 30 µm mesh size with stained pollen sediment were then put on a glass slide with a drop of 50% glycerol (v/v%) aqueous solution. Each slide was scanned with a slide scanner (super COOLSCAN 9000 ED; Nikon®, Japan) by photographing each piece of nylon membrane of 30 µm mesh size independently (Supplementary Figure S6).

Analysis of images of stigmatic pollen loads recovered on nylon membranes

To decrease the large amount of time needed to visually count the stained pollen grains deposited on nylon membranes, we analysed the images of the nylon membranes with stained pollen sediment with the ImageJ software (Schneider *et al.*, 2012), as it had already been used for counting pollen grains (Costa and Yang, 2009; Tello *et al.*, 2018).

To count the number of pollen grains on each image (Supplementary Figure S6), we used the software ImageJ with the plugins *Threshold Colour* (https://blog.bham.ac.uk/intellimic/g-landini-software/) and *Nucleus Counter* implemented in the *Particle Analysis* plugin as part of

the collection *MBF "ImageJ for Microscopy"* (https://imagej.nih.gov/ij/plugins/mbf/index.html; Collins, 2007), uploaded in the plugin file of ImageJ. The procedure started by opening an image and selecting 'Plugins' < 'Threshold Colour' to adjust the 'hue' on the range 110-200. The image was then converted in an 8-bit format by selecting 'Image' < 'Type' < '8-bit'. The image was then cleared by selecting 'Image' < 'Adjust' < 'Threshold…' to select the appropriate spectrum, by shifting the left border to reach the beginning of the spectrum and adjusting the right border so as to keep only the pollen grains appearing in red, before selecting 'Apply' < 'OK'. The appropriate particle size was then chosen by selecting 'Plugins' < 'Nucleus Counter', completing 'Smallest size' with '18', 'Largest size' with '80', 'Threshold method' with 'current', 'Smooth method' with 'none', ticking all the boxes and by selecting 'OK'. The number of counted pollen grains appeared in 'Summary of Threshold' < 'Count'.

References

Collins TJ. 2007. ImageJ for microscopy. *Biotechniques*, *43*(1), 25-30.

Costa CM, and Yang S. 2009. Counting pollen grains using readily available, free image processing and analysis software. *Annals of Botany*, *104*(5), 1005-1010.

Dafni A. 1992. Pollen and stigma biology. In: Dafni A (ed.), *Pollination Ecology: A Practical Approach*. Oxford University Press, pp. 59-89.

Dafni A, Pacini E, and Nepi M. 2005. Pollen and stigma biology. In: Dafni A, Kevan PG, and Husband BC (eds.), *Practical Pollination Biology*. Enviroquest, pp. 83-146.

Schneider CA, Rasband WS, and Eliceiri KW. 2012. NIH Image to ImageJ: 25 years of image analysis. *Nature Methods*, *9*(7), 671-675.

Tello J, Montemayor MI, Forneck A, and Ibáñez J. 2018. A new image-based tool for the high throughput phenotyping of pollen viability: evaluation of inter- and intra-cultivar diversity in grapevine. *Plant Methods*, *14*(1), 3.

Vaissière BE. 1991. Measure of pollination intensity and associated parameters. In: *Honey bees, Apis mellifera L. (Hymenoptera: Apidae), as pollinators of upland cotton, Gossypium hirsutum L. (Malvaceae), for hybrid seed production*. Philosophical Dissertation, Texas A&M University, College Station, Texas, USA, pp. 22-95.

Vaissière BE, and Froissart R. 1996. Pollination of cantaloupes under spun bonded row cover by honey bees (Hymenoptera: Apidae) in West Africa. *Journal of Economic Entomology*, *89*(5), 1213-1222.

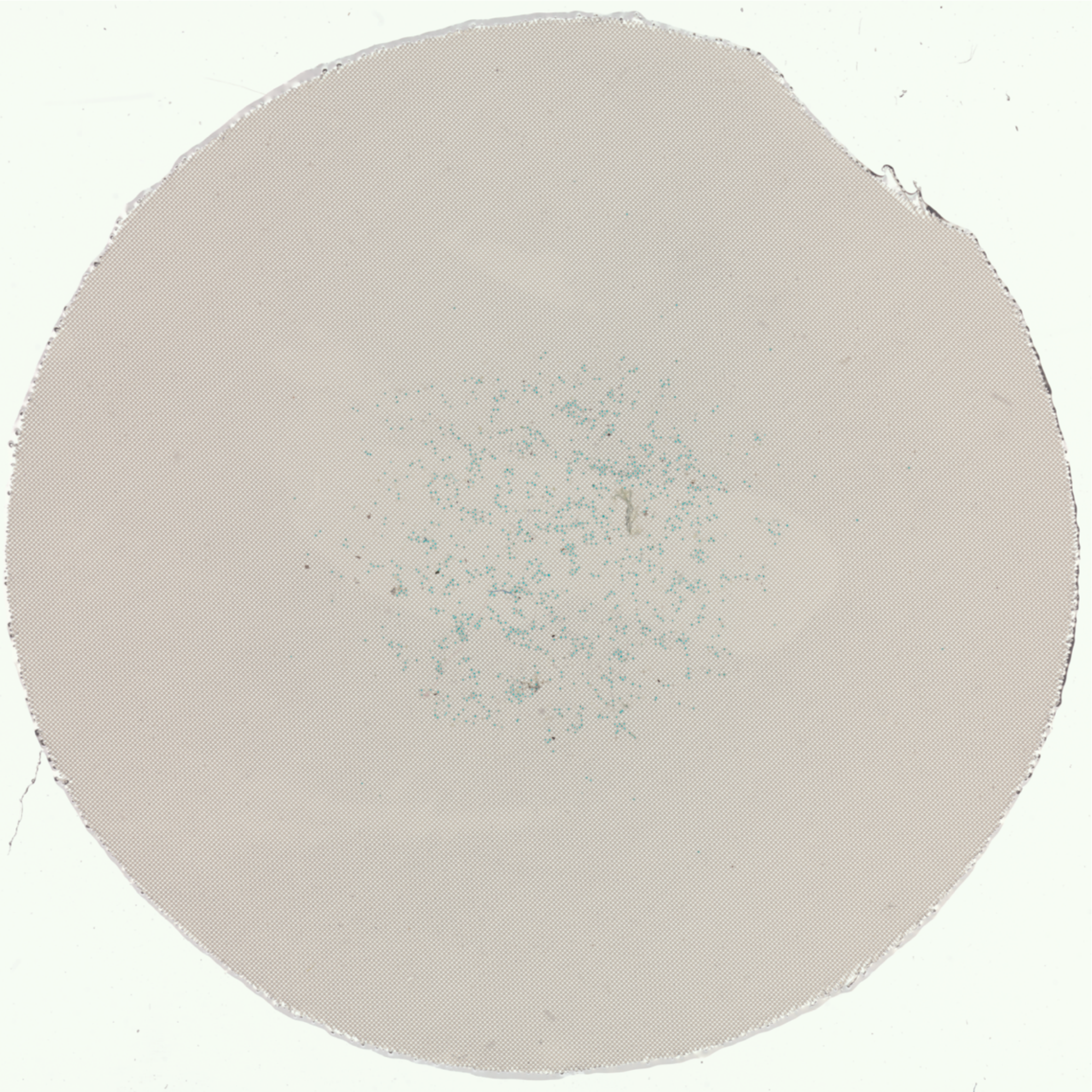

Supplementary Figure S6. Example of an image of melon pollen stained with malachite green and scattered on a piece of nylon membrane of 30 µm mesh size following its extraction from a stigma by an ultrasonic probe. Membrane diameter: Ø25 mm. Photo credit: Marie-Josée Buffière/INRAE, Stan Chabert/INRAE and Géraud de Premorel/INRAE.
